# Supplementary material for: Household food insecurity and its association with self-reported male perpetration of intimate partner violence: a survey of two districts in central and western Uganda
Source: BMJ Open. 2021 Mar 31;11(3):e045427. doi: 10.1136/bmjopen-2020-045427 (PMC8016075; doi:10.1136/bmjopen-2020-045427)
Supplement: Supplementary data [file bmjopen-2020-045427supp001.pdf]

Online supplementary Appendix1:

Sensitivity analysis: Characteristics of the analysed sample (N=1314) and non-responders (N=321)

Variable	Analysed sample N (%)	Non-responders N (%)	p-value
**Study Strata**			
Wakiso communities	**578**	**179**	0.364
**Urban -Kazo**	206(35.6)	68(37.9)	
**Semi-urban -Lukwanga**	219(37.9)	62(34.6)	
**Rural -Sentema**	153(26.5)	49 (27.4)	
Hoima communities	**736**	**142**	0.210
**Urban –Ishaka_Kijungu**	243(33.0)	67(47.2)	
**Semi-urban-Butem**	198(25.5)	39(27.5)	
**Rural -Kitoba**	295(40.1)	42 (29.6)	
**Age (mean, SD), interquartile range(years)**	34 ±12.9, 24-42	15.8 ±3.4, 14-17	<0.05
**Education**			
**Primary school and below**	688(52.3)	149(46.4)	>0.001
**Secondary school**	477(36.3)	111(34.5)	
**Post-secondary**	149(11.3)	61(19.0)	
**Not married**	151(11.5)	0	
**Religion**			
**Christianity**	1068(81.3)	230(71.7)	0.02
**Non-Christians**	246(18.7)	91(28.3)	
**HIV status from blood sample**			
**Negative**	1224(94.3)	310(96.6)	<0.001
**Positive**	74(5.7)	11(3.4)	
**Past-year food insecurity**			
**No food insecurity**	995(75.7)	240(74.9)	0.891
**Low food insecurity**	272(20.7)	74(23.2)	
**High food insecurity**	47(3.6)	7(1.9)	
