# Supplementary material for: Household food insecurity and its association with self-reported male perpetration of intimate partner violence: a survey of two districts in central and western Uganda
Source: BMJ Open. 2021 Mar 31;11(3):e045427. doi: 10.1136/bmjopen-2020-045427 (PMC8016075; doi:10.1136/bmjopen-2020-045427)
Supplement: Author's manuscript [file bmjopen-2020-045427.draft_revisions.pdf]

BMJ Open is committed to open peer review. As part of this commitment we make the peer review history of every article we publish publicly available.

When an article is published we post the peer reviewers' comments and the authors' responses online. We also post the versions of the paper that were used during peer review. These are the versions that the peer review comments apply to.

The versions of the paper that follow are the versions that were submitted during the peer review process. They are not the versions of record or the final published versions. They should not be cited or distributed as the published version of this manuscript.

BMJ Open is an open access journal and the full, final, typeset and author-corrected version of record of the manuscript is available on our site with no access controls, subscription charges or pay-per-view fees (http://bmjopen.bmj.com).

If you have any questions on BMJ Open's open peer review process please email info.bmjopen@bmj.com

BMJ Open

Household food insecurity and its association with Self-reported male perpetration of intimate partner violence: a survey of two districts in Central and Western Uganda

Journal:	*BMJ Open*
Manuscript ID	bmjopen-2020-045427
Article Type:	Original research
Date Submitted by the Author:	06-Oct-2020
Complete List of Authors:	Awungafac, George; Karolinska Institute, Department of Global Public Health<br>Mugamba, Stephen; Africa Medical and Behavioural Science Organization<br>Nalugoda, Fred; Africa Medical and Behavioural Science Organization<br>Sjöland, Carl Fredrik; Karolinska Institute, Department of Global Public Health<br>Kigozi, Godfrey; Africa Medical and Behavioural Science Organization<br>Rautiainen, S; Karolinska Institute, Department of Global Public Health<br>Malyabe, Robert; Africa Medical and Behavioural Science Organization<br>Ziegel, Leo; Karolinska Institute, Department of Global Public Health<br>Nakigozi, Gertrude; Africa Medical and Behavioural Science Organization<br>Nalwoga, Grace; Africa Medical and Behavioural Science Organization<br>Kyasanku, Emmanuel; Africa Medical and Behavioural Science Organization<br>Nkale, James; Africa Medical and Behavioural Science Organization<br>Watya, Stephen; Africa Medical and Behavioural Science Organization<br>Ekström, Anna; Karolinska Institute, Department of Global Public Health<br>Kågesten, Anna; Karolinska Institute, Department of Global Public Health
Keywords:	Public health < INFECTIOUS DISEASES, Epidemiology < INFECTIOUS DISEASES, HIV & AIDS < INFECTIOUS DISEASES

SCHOLARONE™
Manuscripts

I, the Submitting Author has the right to grant and does grant on behalf of all authors of the Work (as defined in the below author licence), an exclusive licence and/or a non-exclusive licence for contributions from authors who are: i) UK Crown employees; ii) where BMJ has agreed a CC-BY licence shall apply, and/or iii) in accordance with the terms applicable for US Federal Government officers or employees acting as part of their official duties; on a worldwide, perpetual, irrevocable, royalty-free basis to BMJ Publishing Group Ltd ("BMJ") its licensees and where the relevant Journal is co-owned by BMJ to the co-owners of the Journal, to publish the Work in this journal and any other BMJ products and to exploit all rights, as set out in our licence.

The Submitting Author accepts and understands that any supply made under these terms is made by BMJ to the Submitting Author unless you are acting as an employee on behalf of your employer or a postgraduate student of an affiliated institution which is paying any applicable article publishing charge ("APC") for Open Access articles. Where the Submitting Author wishes to make the Work available on an Open Access basis (and intends to pay the relevant APC), the terms of reuse of such Open Access shall be governed by a Creative Commons licence – details of these licences and which Creative Commons licence will apply to this Work are set out in our licence referred to above.

Other than as permitted in any relevant BMJ Author's Self Archiving Policies, I confirm this Work has not been accepted for publication elsewhere, is not being considered for publication elsewhere and does not duplicate material already published. I confirm all authors consent to publication of this Work and authorise the granting of this licence.

For peer review only

Household food insecurity and its association with Self-reported male perpetration of intimate partner violence: a survey of two districts in Central and Western Uganda

George Awungafac[1*], Stephen Mugamba[2], Fred Nalugoda[2], Carl Fredrik[1], Godfrey Kigozi[2], Susanne R. Lagerström[1], Robert Bulamba[2], Leo Ziegel[1], Gertrude Nakigozi[1], Grace Kigozi Nalwoga[2], Emmanuel Kyasanku[2], James Nkale[2], Stephen Watya[2], Anna Mia Ekström[1,3], Anna E. Kågesten[1]

Author affiliations

1. Department of Global Public Health, Karolinska Institutet, Stockholm Sweden
2. Africa Medical and Behavioural Sciences Organisation, Kampala, Uganda

*Corresponding author: **awungafacg@yahoo.com**

peer review only

Abstract

Introduction: Evidence of male perpetration of intimate partner violence (IPV) and its potential link with food insecurity can inspire policies on prevention and response in sub-Saharan Africa. This study sought to determine the lifetime prevalence of male perpetrated IPV and to assess its association with food insecurity in rural, urban and semi-urban communities of Uganda.

Methods: A population-based household survey was conducted among 13-80-year-old male residents of Wakiso and Hoima districts, from May 2018 to July 2019 (N=2014). The current study included participants who reported ever being in a sexual union and responded to the IPV questions (N=1314). Outcome categories included no physical and/or sexual IPV, physical versus sexual violence only, and both physical and sexual violence. Food insecurity was measured using standard questions and categorized into no, low and high. Multinomial logistic regression was used to determine the crude and adjusted relative risk ratios (aRRR) of IPV perpetration in relation to self-reported food insecurity and covariates.

Results: The prevalence of self-reported lifetime IPV was 14.6% and 6.5% for physical versus sexual violence only, respectively, and 5.3% of males reported to have perpetrated both physical and sexual IPV. Males 25-34 years comparatively reported the highest prevalence of both physical and sexual violence ($p<0.001$). In adjusted models, food insecurity was associated with increased risk of having perpetrated both physical and sexual violence (aRRR=2.57, 95%CI; 1.52-4.32). Number of sexual partners, having living children, being unmarried and alcohol consumption were also associated with IPV perpetration whereas no association was found for education level, age and religion.

Conclusion: The prevalence of male IPV perpetration in these communities varies by age group, and is most commonly reported by young men. Our results suggest that food insecurity

For peer review only

may be one of the main drivers of IPV perpetration, and this could worsen with the SARS-CoV-2 outbreak.

Key words: Intimate Partner Violence, Men, Food insecurity, Uganda, HIV

Strengths and limitations of the study

- This is the first study to investigate lifetime perpetration of intimate partner violence, and to explore food insecurity as a determinant of IPV in Uganda.
- This study recruited a population-based, diverse and representative sample of participants from all societal levels (rural, semi-urban and urban) in Uganda.
- The cross-sectional nature of the study makes it impossible to establish a temporary association between IPV and food insecurity.
- Asking self-reports of IPV from men may be subject to under-reporting as some men may have considered self-reporting sensitive and shameful.

For peer review only

Introduction

Intimate partner violence (IPV) is a major global health problem and a human rights violation. IPV consists of intentional and abusive attitudes in the form of physical, sexual and/or emotional offense and controlling behaviours within an intimate partnership,(1). While IPV can take different forms, male perpetrated IPV is the most common form of violence against women (VAW),(2). Globally, one in three ever-partnered women report experiencing some form of physical and/or sexual violence in their lifetime, with the WHO AFRO (World Health Organisation's Region for Africa) having the highest prevalence (37%), followed by the WHO Region for the Americas (29.8%),(4). The WHO multi-country study on VAW in the Asia and the Pacific estimated that the prevalence of male IPV perpetration was 21.1% for physical violence only, 11.7% for sexual violence only, and 11.8% for both physical and sexual violence,(5). Reports from diverse social and cultural contexts have demonstrated that IPV perpetration begin early in the life course, with most perpetrators reporting that they first did so during adolescence,(6).

IPV carries multiple and well-established consequences for women's health and wellbeing, with femicide being the most extreme form,(7,8). IPV is associated with an increased risk of HIV and STI acquisition,(9,10), severe depressive symptoms,(11), as well as substance use and abuse, including binge drinking,(11). Furthermore, children born to women experiencing IPV are at higher risk of premature death, poor health outcomes, and emotional and behavioural problems later in life,(12). Experiencing IPV has also been shown to affect women and girls' social and economic empowerment,(14), including decrease in productivity at work, loss of employment opportunities and other important social engagement,(15).

Uganda has one of the world's highest burden of IPV, wherefore women's experiences has taken most of the attention of research on violence against women in the country. The 2014

For peer review only

Ugandan Demographic and Health Survey (UDHS) found that 47% of married women had experienced physical IPV in their lifetime, and 29% reported lifetime sexual IPV,(17). Slapping, hitting and beating a pregnant woman has been found to be common, and shown to worsen during the course of pregnancy in Northern Uganda,(18). The country's HIV incidence has further been found to correlate with the frequency of exposure to, and the duration of experiencing any IPV,(19).

In this paper, we turn our attention to men who are perpetrating IPV. With the exception of Kampala, little is known about male IPV perpetration in different urban, semi-urban and rural segments of the Ugandan society, especially those outside the capital city. While multiple intersection factors drive men's use of violence against women, there is growing evidence that IPV might be linked with food insecurity,(20–22) defined as "a household-level economic and social condition of limited or uncertain access to adequate food",(22). For example, a cross-sectional study in slums of Kampala found that extreme physical violence was common among a convenience sample of young men,(14-24 years), and found to correlate with reported hunger and alcohol consumption,(23). Most evidence, however, comes from other countries including Nepal, South Africa and the United States where studies have established a significant relationship between food insecurity and both IPV experiences among women,(24,25) and IPV perpetration by men,(20). According to the United Nations (UN), the food insecurity situation is worsening in Uganda, increasing from 24.1% in 2006 to 41% in 2018,(26). With the ongoing SARS-CoV-2 outbreak, food insecurity could further worsen in line with predictions of the World Bank and the World Food Programme,(27,28).

Male IPV perpetration against women is a complex multi-level social, economic and structural problem that is rooted in unequal gender norms and systems of power. In most cultural settings, a gendered order exists that favour stereotypically masculine men's dominance over women, as

For peer review only

well as over other, more marginalised masculinities that do not live up to the norms of being a "real" man,(29). IPV can thus be viewed both as a symptom of, and a tool to achieve, this type of "hegemonic" masculinity and power,(30). Being unable to provide food for oneself or the household could be interpreted as failing to meet stereotypical masculinity norms, thereby forcing men to use alternative strategies such as violence to demonstrate their manhood,(20). Mental health issues (depression or anxiety), which occurs when concerns about food availability are accompanied by poor coping mechanisms such as alcohol consumption, can further lead to IPV, as demonstrated by Hatcher and colleagues in peri-urban South Africa,(20). Furthermore, the concept of "patriarchal risk",(31) can help to theoretically explain the cultural and societal dependence of women on male family members for food and protection. The consequence of this phenomenon is that boys may eat bigger portions than girls; resulting in males being prioritized to eat when there is not enough food.

Given the increasing reports of food insecurity in Uganda, it is important to investigate its possible links with male perpetrated IPV as no previous studies have examined this association in low-and-middle income sub-Saharan African settings. Using a representative sample from urban, semi-urban and rural communities in Uganda, the current study seeks to determine the prevalence of self-reported male perpetration of IPV, and to assess its association with food insecurity, controlling for social-demographics and HIV-risk behaviours. The evidence gained can help inform programmes and policies to prevent and better respond to IPV in the country and other similar settings.

For peer review only

Methods

Study design

Data were collected between May 2018 and July 2019 as part of a cross-sectional baseline survey of a longitudinal open population-level cohort established by the Africa Medical and Behavioural Sciences Organization (AMBSO) to conduct population health surveillance (PHS) in the Wakiso and Hoima districts of Uganda. Briefly, the AMBSO PHS is aimed at generating evidence-based data to inform policy on the health status of the population through periodic monitoring of disease trends and determinants of health. Beyond the IPV module used for this study, the survey also collects information including non-communicable diseases (diabetes, hypertension, cancers, etc.), communicable diseases (such as HIV/AIDS and STIs), food and nutrition, health behaviour risks and mental health.

Study communities

With a population of nearly 2 million, the Wakiso district is situated in the Central Region of Uganda and surrounding parts of Kampala, while the Hoima district has a smaller population of about 570,000 and located in the Western Bunyoro Region. The selection of these two districts was purposive, based on their common and diverse characteristics. Wakiso has a high migrant worker population and known to be a hotspot for sex workers and men who have sex with men. Hoima around Lake Albert is rich in oil, and the mining activities there attract migrant workers, sex workers and fisher folks – populations that have high risk of HIV according to previous evidence,(39). Each district was stratified into urban, semi-urban and rural areas. Residents in all urban and semi-urban areas, and those in a randomly selected rural community (representing the rural sub-counties), were involved in the baseline survey.

For peer review only

Study population

The study population comprised of males aged 13-80 years in the study communities. A lower age limit of 13 years was considered because of the high prevalence of early sexual debut in Uganda,(33). This study sample was defined as males who have ever had a sexual relationship. In each study community, the sampling frame consisted of all the households. In every household all males aged 13-80 years were considered potential participants. A minimum sample of N=738 was needed to estimate the associations in the current study with 80% power at the alpha=0.05 level. To be included in the study, participants had to be able and willing to respond to the survey questions and provide informed consent. With the exception of emancipated minors (under 18 years) who (like adults) gave their own written consent, parents/guardians provided written consent for males under 18 years, while verbalassent was also obtained from the minors. The full survey sample consisted of 2014 male participants who were recruited into the AMBSO population health surveillance cohort. Males who reported not to have ever been in a sexual union (N=379) and those who abstained from responding to questions on male IPV perpetration (N=321) were excluded. Even though the individual response rate was 80.4%, the final analysis sample (N=1314) was closed to double the estimated sample of N=738 participants needed to estimate the association. This 80.4% response rate is similar to that of a multi-country survey on male IPV perpetration in Asia,(34). A sensitivity analysis comparing the characteristics of those who responded to the IPV questions and those who did not respond is included in online supplementary appendix 1. The comparison showed that the proportion of the non-responders was similar across the different study communities, but statistically smaller in terms of their age groups (mostly young males were non-responders), religion (non-responders were mostly Christians) and educational levels (majority of non-responder were males with primary education or below). This did not affect the

For peer review only

findings considering that the final sample was more than double the minimum estimated sample to establish the association.

Procedures

Data were collected using a structured questionnaire with ten sections, including: sociodemographics, first sex experience, family planning practice, marital status and practices, food supply and food security, intimate partner violence, HIV testing services (HTS), HIV care and treatment services, prostate cancer and behaviour risk. During each planned visit, trained data collectors of the male sex interviewed male participants face-to-face in private locations around the household. The IPV questions were adapted from the WHO multi-country study on violence against women and domestic violence (35), and translated into the local languages (Luganda in Wakiso and Runyoro-Rutoro in Hoima). Consenting males were asked about their lifetime involvement in IPV perpetration. This study did not consider questions on emotional violence and controlling behaviours due to the potentially more subjective nature of their responses. In addition to these survey questions, the PHS also collects 10 millilitres of venous blood samples that were used to determine the HIV and syphilis status of participants, and aliquots stored for other future studies. HIV status was determined following the Ugandan Ministry of Health (MOH) rapid test algorithm(36), and the rapid plasma reagin serological (RPR) test was used to determine syphilis status of the participants.

Dependent variables

The dependent variable, lifetime perpetration of IPV, was defined as lifetime self-reported perpetration of physical and/or sexual violence against an intimate (sexual) partner, and grouped into four independent and unordered categories: 1) no perpetration of physical and/or sexual violence (no IPV); 2) physical violence only; 3) sexual violence only; and 4) both physical and

For peer review only

sexual violence. These definitions and the WHO coding,(30) are illustrated in Table 1. The "no IPV perpetration" category was used as the reference group, referring to males reporting to never have perpetrated physical and/or sexual violence against their intimate sexual partner. The "physical violence only" category consisted of males reporting to have perpetrated one or more forms of physical violence, but had never used any sexual violence; "sexual violence only" was coded using the same approach but referring to one or more forms of sexual violence. Males who reported to have perpetrated both physical and sexual violence in their lifetime were grouped into the "physical and sexual violence" category.

Table 1. Definition and measurement of lifetime IPV perpetration (dependent variable)

Type of violence perpetration	**Have you ever done any of the following to your sexual partner?**
**Physical violence only (“yes” to ≥1 indicator)**	- Push, slap or hold partner down?<br>- Punch with fist or with something that could hurt partner?<br>- Kick or drag partner?<br>- Tried to strangle or burn partner?<br>- Attacked partner with a knife, gun, or other type of weapon?
**Sexual violence only (“yes” to ≥1 indicator)**	- Force her to perform sexual acts against partner’s will?<br>- Used threats to force her to have sex when she did not want to?<br>- Physically force her to have sex against her wish?
**Physical and sexual violence (“yes” to ≥1 indicators of both)**	- Perpetration of both physical violence and sexual violence
**No IPV perpetration**	- No perpetration of both physical violence and sexual violence

Independent variables

Food insecurity measurement

The main independent variable was food insecurity, assessed using an adapted version of the Food Insecurity Experience Scale (FIES) questions,(37) in which six selected items (online supplementary appendix 2) were used to evaluate the household food situation based on self-reports of experiences during the preceding 12 months. In line with international definitions,(38), negative responses to assessment questions such as "no" "never" "only 1 or 2 months" were

For peer review only

coded as “0”, (referring to those who reported having minor access problems for food during the preceding 12 months) while positive or affirmative responses including “yes”, “always”, “almost every month”, “some months but not every month” were coded as “1” (referring to having problems with access to adequate food during the preceding 12 months). All codes were summed up to obtain a total score for food insecurity, with higher score indicating the extent of food insecurity. As adapted from international approaches (38), participants were categorised according to scores as: no food insecurity (0-1), low food insecurity (2-4) and high food insecurity (hunger) (5-6). Similar to previous studies,(20), low food insecurity and high food insecurity were merged during modelling due to the ue to the small proportion reporting high food insecurity.

Covariates

Sociodemographic variables included study district (urban/semi-urban/rural); age; education level (categorized into primary school and below, secondary school and post-secondary), marital status (married or in a union/not married); religion (categorized into Christian, non-Christians); and having living children (yes/no). HIV-risk behaviours were considered among the covariates, and included, number of sexual partners (one partner/two or more partners), condoms use past year (yes/no), use of illicit drugs (yes/no), alcohol consumption (yes/ no) and male circumcision (yes/no).

Statistical analysis

We first conducted descriptive analyses to explore the distribution in variables and check for outliers. Missing values for covariates were replaced using multiple imputation assuming random missingness. Pearson’s Chi-squared test and Fishers exact test were used to compare proportions with α set at 5%. Next, we performed bivariate multinomial logistic regression (MLR)

For peer review only

to determine the crude relative risk ratios (RRR) and 95% confidence intervals (CI) of IPV perpetration in relation to the independent variable and covariates. We followed the approach used by Fulu et al,(5) and used “No IPV perpetration” as the base outcome against which all other outcomes were compared. This type of regression produces RRR, which are the exponentiated coefficients of the regression, and explain the relative effect of the independent variable on the outcomes using one of the outcomes as a base outcome. A full model was built to explain the associated factors to IPV perpetration in relation to both food insecurity, HIV-risk behaviours and sociodemographic background. The full model comprised of all the study variables. The final model comparing associated factors to IPV perpetration was selected using maximum likelihood ratio (LR) test. All analyses were conducted using STATA version 16 (StataCorp) while Microsoft Office Excel was used to design figures.

Ethical considerations

The AMBSO PHS cohort study received ethical approval from the Clarke International University Ethics Committee (Ref:CIUREC/0059) as the local Institutional Review Board (IRB) of record, and clearance from the Ugandan Council of Science and Technology (Ref:SS4468). Clearance was obtained from both districts to conduct the sudy. All participants provided a written informed consent /assent to participate in the study. Data was collected by a study team trained and certified in Human subject protection and Good clinical Practice. Interviews were conducted in private, unique identification codes were used in all computerized data, and the lists that linked participants’ names to the codes were accessible only to senior project staffs in password protected computers. Participants who tested positive for HIV infection and syphilis were linked to specialized care while those who needed other health support services were referred to health facilities of their choice.

For peer review only

Patients and Public Involvement statement

The study team worked hand in hand with the community health workers, local community leaders and a community-research advisory structure to develop and clarify a common underdstanding of the aims of the polulation-based survey, the designs, operational aspects of the survey and how results will be disseminated. As part of this “patient and public involvement” effort, a consensus was reach with the advisory committee on a plan to provide a general public awareness about the study and on health issues which were planned to be the areas of focus of the PHS.

Results

Respondents’ characteristics

Table 2 shows the characteristics of the study participants. The mean age of respondents was 34 years (SD; ±12.9). Food insecurity was found to affect one in four men, with 20.7% and 3.6% categorized as low versus high food insecurity, respectively. About one third and 50% of men reported having had multiple sex partners and used alcohol in the past year respectively; and approximately 6% tested positive for HIV.

Table 2. Sample characteristics of male respondents in a survey of lifetime IPV perpetration in two districts of Uganda (N=1314) from May 2018 to July 2019

Variable	n	%
Age (mean, SD)	34 ±12.9	
Education		
Primary and below	688	52.3
Secondary school	477	36.3
Post-secondary	149	11.3
Marital status		
Married or in a union	1163	88.5
Not married	151	11.5
Have any living children		
Yes	999	76.2
No	312	23.8

For peer review only

Religion		
Christianity	1068	81.3
Non-Christians	246	18.7
Past-year food security		
No food insecurity	995	75.7
Low food insecurity	272	20.7
High food insecurity	47	3.6
Score (mean, SD)	1.48 ±1.05	
Number of lifetime sexual partners		
One partner	919	69.9
Two or more partners	395	30.1
Current use of condoms		
Yes	519	39.5
No	795	60.5
HIV status from blood sample		
Negative	1224	94.3
Positive	74	5.7
Past-year alcohol use		
Yes	677	48.5
No	640	51.5
Circumcised		
Yes	659	50.2
No	655	49.8
Past-year use of illicit drugs		
Yes	43	3.3
No	1271	96.7

Prevalence of lifetime IPV perpetration

Table 3 presents data on the prevalence of perpetration of different forms of IPV against an intimate partner by male participants in the survey. Most (73.6%) males reported to never have perpetrated any form of IPV in their lifetime. The prevalence of self-reported physical IPV ranged from 19.3% (pushed, slapped, held down) to 0.9% (attacked partner with a knife, gun or other weapon, with 14.6% having perpetrated any form of physical violence only. Lifetime perpetration of sexual IPV ranged from 8.2% (threatened or pressured partner into sex when unwanted) to 5.4% (physically forced partner to have sex) and 2.4% (used other types of force to have sex), with 6.5% of males reporting to have perpetrated any form of sexual IPV only. In total, 5.3% of males reported to have perpetrated both physical and sexual IPV.

For peer review only

Table 3: Prevalence of self-reported lifetime perpetration of IPV against a sexual partner among ever-partnered males aged 13-80 years in two districts of Uganda, May 2018 to July 2019

	**Lifetime IPV perpetration (N=1314)**	
	n	%
**Physical violence (any form)**	261	19.9
Push, slapped or held down partner	253	19.3
Kicked or dragged partner	54	4.1
Tried to strangle partner	19	1.5
Attacked partner with a knife, gun or other weapon	12	0.9
Punch with fist or with something that could hurt partner?	61	4.6
**Sexual violence (any form)**	155	11.8
Threatened or pressured partner into sex when unwanted	108	8.2
Physically forced partner into sex	70	5.4
Used other ways to force partner to perform sexual acts when unwanted	31	2.4
**Any physical or sexual violence**	347	26.4
**Physical IPV perpetration only**	192	14.6
**Sexual IPV perpetration only**	86	6.5
**Both physical and sexual IPV perpetration**	69	5.3
**No IPV perpetration**	967	73.6

Figure 1 shows the prevalence of self-reported IPV perpetration across different age groups. As can be seen, the prevalence of physical violence only and both physical and sexual violence was highest among 25-34-year-olds compared to the other age-groups ($p<0.001$), with perpetration of sexual violence only being most commonly reported by young males (18-24 years) and less common after age 34 ($p=0.517$).

Factors associated with IPV perpetration

Bivariate analysis

In bivariate analysis, food insecure participants had an RRR of 2.71 (95% CI 1.64-4.46) to have perpetrated both physical and sexual violence, but no significantly higher risk to have perpetrated physical vs. sexual violence only, respectively (Table 4). Compared to the youngest age group 13-24 years, young adult participants' (25-34 years) had a RRR of 2.59 (95%CI 1.64-4.07) for perpetrating physical violence only, and 2.64 (95%CI 1.30-5.36) for both physical and

For peer review only

sexual violence, and the RRR for those aged 35+ years was 2.16 (95%CI 1.38-3.38) for physical violence. Being married was associated with a lower relative risk of having perpetrated sexual violence only. Having living children and reporting no condom use was associated with perpetration of physical violence only. Education level, was not significantly associated with IPV perpetration. Reported use of illisit drugs the past year was associciated with perpetration of physical violence only and both physical and sexual violence.

For peer review only

Table *4*: Crude Relative Risk Ratios (RRR) of male IPV perpetration in relation to sociodemographic factors, food insecurity, HIV-related factors in two districts of Uganda (baseline outcome=no IPV) (N=1314), from May 2018 to July 2019

**Variable**	**N**	**No IPV**	**Physical violence only**		**Sexual violence only**		**Both Physical and sexual violence**	
		N (%)*	N (%)*	RRR, 95% CI	N (%)*	RRR, 95% CI,	N (%)*	RRR, 95% CI,
Age group (ref: 13-24 years)								
25-34	439	304(69.3)	80(18.2)	2.59(1.64-4.07)	24(5.5)	0.80(0.46-1.42)	31(7.1)	2.64(1.30-5.36)
35+ years	522	378(72.4)	83(15.9))	2.16(1.38-3.38)	34(6.5)	0.92(0.54-1.54)	27(5.2)	1.85(0.90-3.79)
Education (Ref: Primary or below)								
Secondary school	477	349(73.2	68(14.3)	0.92(0.66-1.28)	34(7.1)	1.05(0.66-1.68)	26(5.5)	1.0(0.60-1.69)
Post-secondary	149	119(79.9)	18(12.1)	0.71(0.42-1.21)	6(4.0)	0.55(0.23-1.31)	6(4.0)	0.68(0.28-1.65)
Christian religion (Ref: Islam and others)	1072	781(72.8)	168(15.7)	1.66(1.06-2.63)	70(6.5)	1.04(0.59-1.83)	53(4.9)	0.79(0.44-1.41)
**Married (Ref: not married)**	1163	858(73.8)	177(15.2)	1.50(0.85-2.63)	68(5.9)	0.48(0.27-0.84)	60(5.2)	0.85(0.41-1.75)
Have living children (Ref: no)	999	708(70.9)	172(17.2)	3.48(2.10-5.77)	61(6.1)	0.89(0.54-1.45)	58(5.8)	1.92(0.99-3.71)
Drink alcohol (Ref: no)	677	456(67.4)	118(17.4)	1.79(1.30-2.45)	54(7.9)	1.89(1.20-1.98)	49(7.3)	2.74(1.61-4.68)
Past year illicit drug (Ref: no)	43	19(44.2)	12(27.9)	3.32(1.59-6.97)	3(6.9)	1.80(0.52-1.22)	9(20.9)	7.48(3.24-17.2)
**Food insecure (Ref: no food insecurity)**	319	214(67.1)	52(16.3)	1.31(0.92-1.86)	23(7.2)	1.28(0.77-1.22)	30(9.4)	2.71(1.64-4.46)
Food insecurity score				1.08(0.94-1.25)		1.03(0.84-1.27)		1.30(1.09-1.55)
Condom use in past 12 months (Ref: no)								
Yes	519	396(76.3)	62(11.9)	0.69(0.49-0.96)	36(6.9)	1.04(0.66-1.62)	25(4.8)	0.82(0.49-1.36)
**Circumcised (Ref: no)**	659	503(76.3)	85(12.9)	0.73(0.54-1.00)	43(6.5)	0.92(0.59-1.43)	28(4.3)	0.63(0.38-1.03)
Two or more lifetime sexual partners (Ref: one)	395	266(67.3)	68(17.2)	1.44(1.04-2.01)	31(7.8)	1.48(0.94-2.36)	30(7.5)	2.03 (1.23-3.33)
Hoima district (Ref: Wakiso)	736	526(71.5)	112(15.2)	1.17(0.86-1.61)	55(7.5)	1.49(0.94-2.35)	43(5.8)	1.38 (0.84-2.29)

*Row percentage

For peer review only

Multivariable analysis

Table 5 illustrates the adjusted relative risk (aRRR) for IPV perpetration in relation to food insecurity adjusted for sociodemographic factors and HIV-risk behaviours. Food insecurity remained significantly associated with self-reported perpetration of both physical and sexual violence (aRRR=2.57, 95%CI; 1.52-4.32). Having two or more sexual partners also remained associated with perpetration of physical violence only (aRRR 1.64, 95%CI; 1.15-2.33) and both physical and sexual violence (aRRR=1.97, 95%CI; 1.15-2.37). Alcohol consumption remained associated with all typologies of IPV perpetration under study. Reported use of illicit drugs remained associated with physical violence and both physical and sexual violence. Having living children was associated with perpetration of physical only, while being married appeared to be protective against perpetration of sexual violence only. Age and religion were not associated with any IPV perpetration in the adjusted model.

Table 5: Final model showing adjusted relative risk ratios (aRRR) of male IPV perpetration in relation to food insecurity and HIV-related factors in two districts of Uganda

variable	Physical violence only*		Sexual violence only*		Both physical and sexual violence*	
	Crude RRR, 95% CI	aRRR, 95% CI	Crude RRR, 95% CI	aRRR, 95% CI	Crude RRR, 95% CI	aRRR, 95% CI
Food-insecurity (ref: no food insecurity)	1.31 (0.92-1.86	1.19 (0.82-1.73)	1.28 (0.77-2.12	1.33 (0.54-1.55)	2.71 (1.64-4.46)	2.57 (1.52-4.32)
Two or more sexual partners (ref: one partner)	1.0 (1.04-2.01)	1.64 (1.15-2.33)	1.48 (0.94-2.36)	1.38 (0.85-2.25)	2.03 (1.23-3.33)	1.97 (1.15-3.37)
Drink alcohol (ref: no)	1.79 (1.30-2.45)	1.51 (1.07-2.12)	1.89 (1.20-2.98)	1.89 (1.16-3.07)	2.74 (1.61-4.68)	2.55 (1.42-4.58)
Illicit drug use the past year (ref: no)	3.32 (1.59-6.97)	3.84 (1.73-8.52)	1.80 (0.52-6.22)	1.29 (0.36-4.58)	7.48 (3.24-17.2)	4.80 (1.93-11.9)
Sociodemographic covariates						
25-34 years (ref: 18-24 years)	2.59 (1.64-4.07)	1.37 (0.99-1.91)	0.80 (0.46-1.42)	0.84 (0.51-1.39)	2.64 (1.30-5.36)	1.66 (0.99-2.78)
Christian religion (ref: non-Christians)	1.66 (1.06-2.63)	1.53 (0.94-2.51)	1.04 (0.59-1.83)	0.82 (0.45-1.51)	0.79 (0.44-1.41)	0.61 (0.32-1.17)
Married (Ref: not married	1.50(0.85-2.63)	1.34 (0.76-2.44)	0.48(0.27-0.84)	0.49 (0.27-0.86)	0.85(0.41-1.75)	0.81 (0.37-1.75)
Have children (ref:	3.48	3.24	0.89	0.92	1.92	1.77

For peer review only

no)	(2.10-5.77)	(1.91-5.52)	(0.54-1.45)	(0.55-1.56)	(0.99-3.71)	(0.87-3.61)

*Reference/base outcome: no IPV perpetration.

Discussion

This study revealed that about 1 in 4 men have ever perpetrated any form of violence against their intimate partners in the study setting. The prevalence of male perpetrated IPV ranged from 5.3%-14.6% for combined or one type of violence; physical versus sexual violence only were 14.6% versus 6.5%, respectively; and 5.3% of males reported to have perpetrated both physical and sexual violence.

Comparing the prevalence of lifetime male IPV perpetration found in our study with data from previous studies is complicated, mainly due to the limited research into the problem, and the non-standardized ways in which surveys assess male IPV perpetration. For instance, even though the 2006 Uganda demographic and health survey (DHS),(39) found a prevalence of 40% for males who perpetrate IPV against women, this was based only on male reports of some forms of physical violence only such as partner hitting, slapping, and kicking, without taking into account the perpetration of sexual violence. The only other related large-scale study that focused on only past-year perpetration in the Rakai district of Uganda indicated a prevalence of 10.4% for physical violence and 3.1% for sexual violence,(40). In the current study, 19.3% reported ever slapping or pushing their intimate partners, estimates that are similar to other countries including Ghana (17.4%),(41) and Vietnam (23.5%),(42), but lower than those obtained by the International Men and Gender Equality study (IMAGES) in the Democratic Republic of Congo (45.1%) and Rwanda (39.1%),(43), while physical violence only was reported by 27.7% of men in a study from Ghana,(41). Cultural, societal and institutional norms and values surrounding gender roles vary across contexts and probably can explain the

For peer review only

prevalence of IPV perpetration obtained in this study. Differences in societal views and/or taboos around IPV questions could also affect the estimates.

One in four men were found to be food insecure, and food insecurity was associated with almost three times the relative risk of having perpetrated both physical and sexual violence. This finding is consistent with results of a recent study from a peri-urban setting of South Africa, in which food insecurity doubled the odds of males' perpetration of IPV,(20). The disparity is that the study design in South African study,(20) was longitudinal while the current study is cross-sectional. The findings from our study also concur with results of studies from Nepal,(25) and USA,(24), which found that food insecurity was more common among married women who had experienced physical violence from their intimate sexual partners. In the present context of SARS CoV2 pandemic, food security could be an underliying risk factor of IPV perpetration. Available research indicates that the association between food insecurity and IPV perpetration might be driven by mental health problems such as anxiety and depression arising from concerns about food availability,(20), and the effect of gender norms whereby the inability of men to provide for partners and households affects their perceived masculinity and contributes to their use of violence,(44). For example, a qualitative study among women and men in Bangladesh (45) found that inadequate food portions offered to men may trigger retaliatory acts of violence against their wives, and men could withhold resources used to acquire food as a form of power. After adjusting for food insecurity and sociodemographics, the study concluded that men who consumed alcohol in the past year and have multiple sexual partners had a significantly higher risk of perpetrating IPV, which is in line with other studies,(36,60, 52).

We further found that the prevalence of IPV perpetration varies by age groups, with reports of sexual and physical violence being more common among younger males compared to older men – findings that are in line with previous studies from the US,(47,48). It is possible that a

For peer review only - http://bmjopen.bmj.com/site/about/guidelines.xhtml

relative lack of relationship experiences among young men and gender-related vulnerabilities, constructed by the social and political system could increase the tendency to perpetrate violence. Peer influence has also been linked to IPV perpetration in young people,(47,48). These findings have implications in designing preventive interventions for different sub-populations especially men in the younger age groups.

Our findings underline the need for early prevention of IPV that targets young men, and the necessity to investigate and respond to food insecurity within the framework of IPV prevention. Other factors associated with IPV perpetration by men such as having many sexual partners, alcohol consumption and drug use are modifiable and subject to change following interventions. This study also emphasizes the need to confront violence against women in a multi-faceted manner, as in the planning of existing interventions in Uganda such as the SASA community based program in Kampala,(49). The Program P initiative,(50) provides concrete guiding strategies for IPV programs to use in efforts to engage men in maternal and child health activities, supporting active fatherhood from time of pregnancy till early childhood globally. These initiatives also contain comprehensive strategies including HIV risk reduction, and should be strengthened and scaled up to other parts of the country.

Limitations

This study is one of the first to investigate lifetime perpetration of different forms of physical and sexual violence among both younger and older men, and to examine their association between food insecurity and male violence perpetration in a population-based, representative cohort in rural, urban and semi-urban Uganda. These findings complement existing literature on the experiences of IPV among women in Uganda. However, the findings of this study must be viewed in light of its limitations. Firstly, given the self-reported nature of the data on a sensitive topic like IPV, it is possible that under-reporting IPV and recall bias occurred. Some participants

For peer review only

may have considered the questions as private, criminal and/or anti-social, shameful to talk about, and therefore found it challenging to reveal about it. However, the training of data collectors possibly improved the confidence in self-reporting IPV perpetration by males. Although relevant variables such as income levels or employment status of the males were not analysed, it is possible that the reported food insecurity is a reflection of the income level and the availability and access to livelihood resources. Finally, due to the cross-sectional nature of the data, we were unable to establish a temporal association between food insecurity and IPV perpetration in males.

Conclusions

The prevalence of male IPV perpetration in these communities varies by age group, with higher rates of violence among younger men, which should draw the attention of IPV preventive strategies and programming. Food insecurity should be considered an underlying determinant of violence against women by their intimate sexual partners. It will be essential to consider the integration and strengthening of IPV and HIV-risk reduction interventions, employing strategies that consider the possibility of food insecurity as the driver of vilence in intimate relationships. The possible impact of SARS-CoV-2 should be considered in IPV interventions. Longitudinal surveys are needed to investigate the drivers of food insecurity in Uganda and other low income countries and its impact on different forms of violence.

Contributorship statement

The original ideas leading to the Population Health Surveillance study from which this manuscript is derived, were put together by SM, FN, GK, GN, SW and AME. GA and AEK conceptualised the research questions for the current study. GA analysed the data, wrote the

For peer review only

first draft of the study and oversaw the manuscript development process. SM, FN, SW and GK coordinated the participant recruitment as well as the data collection, management and entry. RB, LZ, GN, GKN, EK and JN participated in actual data collection from the study participants. CF and SRL contributed in describing the measurement and classification of food insecurity. AME and AEK provided oversight to the study, providing edits, structure and scope to the study. All authors reviewed the drafts leading to this final manuscript.

Funding

The funding of this work was jointly supported with private funds from AMBSO directors, the Karolinska Institute through a Swedish Research Council grant (grant number:2019-04474), and the Swedish Institute (SI) which supported the first author with an educational study grant (Ref:02205/2019) that enabled him to participate in leading this study.

Acknowledgement

We thank the staff of Africa Medical and Behavioral Sciences Organization (AMBSO); the PHS study participants; the local community leadership; and the Wakiso and Hoima Districts Directorates of Health services for supporting this work.

Conflict of interest

None declared

For peer review only

Data availability statement

The data for this study is strictly not available to the public. A reasonable request for the data can be done by contacting Stephen Mugamba (**smugamba@gmail.com**)

References

1 EG et al., eds. World report on violence and health. Geneva, World Health Organization, 2002.

2. Richardson JR, Feder G, Coid J. Domestic violence affects women more than men. BMJ Br Med J. 2002;325(7367):779.

3. Swan SC, Gambone LJ, Caldwell JE, Sullivan TP, Snow DL. A Review of Research on Women's Use of Violence With Male Intimate Partners. Violence Vict. 2008;23(3):301.

4. World Health Organisation. Global and regional estimates of violence against women: prevalence and health effects of intimate partner violence and non-partner sexual violence. Geneva; 2013.

5. Fulu E, Jewkes R, Roselli T, Garcia-Moreno C. Prevalence of and factors associated with male perpetration of intimate partner violence: findings from the UN Multi-country Cross-sectional Study on Men and Violence in Asia and the Pacific. Lancet Glob Heal. 2013 Oct 1;1(4):e187–207.

6. Peitzmeier SM, H MSP, Kågesten A, H MP, Acharya R, Ph D, et al. Intimate Partner Violence Perpetration Among Adolescent Males in Disadvantaged Neighborhoods Globally. 2016;1–7.

7. Martin PM, Carvajal N. Feminicide as 'act' and 'process': a geography of gendered violence in Oaxaca. Gender, Place Cult. 2016;23(7):989–1002.

8. Prieto-Carrón M, Thomson M, Macdonald M. No more killings! Women respond to femicides in Central America. Gend Dev. 2007;15(1):25–40.

9. Campbell JC, Baty ML, Ghandour RM, Stockman JK, Francisco L, Wagman J. The intersection of intimate partner violence against women and HIV/AIDS: a review. Int J Inj Contr Saf Promot. 2008;15(4):221–31.

For peer review only

10. Coker AL. Does physical intimate partner violence affect sexual health?: A systematic review. Vol. 8, Trauma, Violence, and Abuse. Sage PublicationsSage CA: Los Angeles, CA; 2007. p. 149–77.

11. Tsai AC, Wolfe WR, Kumbakumba E, Kawuma A, Hunt PW, Martin JN, et al. Prospective Study of the Mental Health Consequences of Sexual Violence Among Women Living With HIV in Rural Uganda. J Interpers Violence. 2015;31(8):1531–53.

12. Hossain MA, Sumi NS, Haque ME, Bari W. Consequences of Intimate Partner Violence Against Women on Under-Five Child Mortality in Bangladesh. J Interpers Violence. 2014;29(8):1402–17.

13. The World Bank. The Cost of Violence. Washington DC; 2009.

14. Ansara DL, Hindin MJ. Psychosocial consequences of intimate partner violence for women and men in Canada. J Interpers Violence. 2011;26(8):1628–45.

15. López-Sánchez MJ, Belso-Martínez JA, Hervás-Oliver JL. A Review of Economic Consequences and Costs of Male Violence Against Women. Indian J Gend Stud. 2019;26(3):424–34.

16. Food and Agricultural Organisation of the United Nation. How can food security interventions contribute to reducing gender-based violence ?Issue Brief. 2017;

17. Ogland EG, Xu X, Bartkowski JP, Ogland CP. Intimate Partner Violence Against Married Women in Uganda. J Fam Violence. 2014 Nov 18;29(8):869–79.

18. Clarke S, Richmond R, Black E, Fry H, Obol JH, Worth H, et al. Intimate partner violence in pregnancy : sectional study from post- - conflict a cross- - northern Uganda. 2019;1–9.

19. Kouyoumdjian FG, Calzavara LM, Bondy SJ, O'Campo P, Serwadda D, Nalugoda F, et al. Intimate partner violence is associated with incident HIV infection in women in Uganda. Aids. 2013;27(8):1331–8.

20. Hatcher AM, Stöckl H, McBride R-S, Khumalo M, Christofides N. Pathways From Food Insecurity to Intimate Partner Violence Perpetration Among Peri-Urban Men in South Africa. Am J Prev Med. 2019 May 1;56(5):765–72.

21. Yikii F, Turyahabwe N, Bashaasha B. Prevalence of household food insecurity in wetland adjacent areas of Uganda. Agric Food Secur. 2017 Dec 27;6(1):63.

22. Food Insecurity: A Public Health Issue. Public Health Rep. 2016;131(5):655.

For peer review only

23. Swahn MH, Gressard L, Palmier JB, Kasirye R, Lynch C, Yao H. Serious violence victimization and perpetration among youth living in the slums of Kampala, Uganda. West J Emerg Med. 2012;13(3):253–9.

24. Ricks JL, Cochran SD, Arah OA, Williams JK, Seeman TE. Food insecurity and intimate partner violence against women: Results from the California Women's Health Survey. Public Health Nutr. 2016;19(5):914–23.

25. Diamond-Smith N, Conroy AA, Tsai AC, Nekkanti M, Weiser SD. Food insecurity and intimate partner violence among married women in Nepal. J Glob Health. 2019 Jun;9(1):010412.

26. Uganda's food insecurity worsens - UN report [Internet]. [cited 2020 Apr 30]. Available from: https://www.independent.co.ug/ugandas-food-insecurity-worsens-un-report/

27. Programme WF. COVID-19 will double number of people facing food crises unless swift action is taken | World Food Programme [Internet]. 2020. Available from: https://www.wfp.org/news/covid-19-will-double-number-people-facing-food-crises-unless-swift-action-taken

28. The World Bank. Food Security and COVID-19 [Internet]. [cited 2020 May 15]. Available from: https://www.worldbank.org/en/topic/agriculture/brief/food-security-and-covid-19

29. Jewkes R, Morrell R. Hegemonic Masculinity , Violence , and Gender Equality : Using Latent Class Analysis to Investigate the Origins and Correlates of Differences between Men. 2018;21(4):547–71.

30. Jewkes R, Morrell R. Gender and sexuality : emerging perspectives from the heterosexual epidemic in South Africa and implications for HIV risk and prevention. 2010;1–11.

31. Cain M, Khanam SR, Nahar S. Class, Patriarchy, and Women's Work in Bangladesh. Popul Dev Rev. 1979 Sep;5(3):405.

32. Kamali A, Nsubuga RN, Ruzagira E, Bahemuka U, Asiki G, Price MA, et al. Heterogeneity of HIV incidence: a comparative analysis between fishing communities and in a neighbouring rural general population, Uganda, and implications for HIV control. Sex Transm Infect. 2016 Sep 1;92(6):447–54.

33. Statistics UB of. Women and Men in Uganda - Facts and Figures 2016. 2017.

34. Fulu E, Rachel P, Mbbs J, Roselli T, Garcia-moreno C, Study UNMC. Prevalence of and factors

For peer review only

associated with male perpetration of intimate partner violence : fi ndings from the UN Multi-country Cross-sectional Study on Men and Violence in Asia and the Pacifi c. Lancet Glob Heal. 2013;1(4):e187–207.

35. World Health Organization. WHO multi-country study on womens health an domestic violence against women. 2005.

36. Uganda. Ministry of Health. 2003. Uganda national policy guidelines for HIV voluntary counselling and testing. Kampala: Ministry of Health.

37. Ballard, T.J., Kepple, A.W. & Cafiero, C. 2013. The food insecurity experience scale: developing a global standard for monitoring hunger worldwide. Technical Paper. Rome, FAO. (available at http://www.fao.org/economic/ess/ess-fs/voices/en/).

38. Bickel, Gary; Nord, Mark; Price, Cristofer; Hamilton, William; Cook J. Guide to Measuring Household Food Security,; prepared by Bickel, Bickel Gary Mark, Nord Cristofer, Price William, Hamilton and John, Cook. 2000;82.

39. Speizer IS. Intimate partner violence attitudes and experience among women and men in Uganda. J Interpers Violence. 2010 Jul;25(7):1224–41.

40. Mullinax M, Grilo SA, Song XS, Wagman J, Mathur S, Nalugoda F, et al. HIV-Risk Behaviors of Men Who Perpetrate Intimate Partner Violence in Rakai, Uganda. AIDS Educ Prev. 2017 Dec;29(6):527–39.

41. Chirwa ED, Sikweyiya Y, Addo-Lartey AA, Ogum Alangea D, Coker-Appiah D, Adanu RMK, et al. Prevalence and risk factors of physical or sexual intimate violence perpetration amongst men in four districts in the central region of Ghana: Baseline findings from a cluster randomised controlled trial. Dalby AR, editor. PLoS One. 2018 Mar 9;13(3):e0191663.

42. Yount KM, Higgins EM, VanderEnde KE, Krause KH, Minh TH, Schuler SR, et al. Men's Perpetration of Intimate Partner Violence in Vietnam. Men Masc. 2016 Apr 24;19(1):64–84.

43. Fleming PJ, Mccleary-sills J, Morton M, Levtov R, Heilman B, Barker G. Risk Factors for Men ' s Lifetime Perpetration of Physical Violence against Intimate Partners : Results from the International Men and Gender Equality Survey (IMAGES) in Eight Countries. 2015;1–18.

44. Cardoso LF, Gupta J, Shuman S, Cole H, Kpebo D, Falb KL. What Factors Contribute to Intimate Partner Violence Against Women in Urban, Conflict-Affected Settings? Qualitative Findings from

For peer review only

Abidjan, Côte d'Ivoire. J Urban Heal. 2016 Apr 21;93(2):364–78.

45. Lentz EC. Complicating narratives of women's food and nutrition insecurity: Domestic violence in rural Bangladesh. World Dev. 2018 Apr 1;104:271–80.

46. Culbreth R, Swahn MH, Salazar LF, Kasirye R, Musuya T. Intimate Partner Violence and Associated Risk Factors Among Youth in the Slums of Kampala. J Interpers Violence. 2019 Nov 29;088626051988992.

47. Mckool M, Stephenson R, Winskell K, Tharp T, Parrott D, Arbor A. Case for a Social Norms Approach. 2019;

48. Swartout KM. The company they keep: How peer networks influence male sexual aggression. Psychol Violence. 2013;3(2):157–71.

49. Abramsky T, Devries K, Kiss L, Francisco L, Nakuti J, Musuya T, et al. A community mobilisation intervention to prevent violence against women and reduce HIV/AIDS risk in Kampala, Uganda (the SASA! Study): study protocol for a cluster randomised controlled trial. Trials. 2012;13:96.

50. Promundo, CulturaSalud, and REDMAS (2013). Program P – A Manual for Engaging Men in Father- hood, Caregiving, Maternal and Child Health. Promundo: Rio de Janeiro, Brazil and Washington, D.C. USA. These materials can be reproduced provided credit is giv

Figure 1: Depiction of lifetime prevalence by age group of male lifetime IPV perpetration in two districts of Uganda, May 2018 to July 2019.

For peer review only

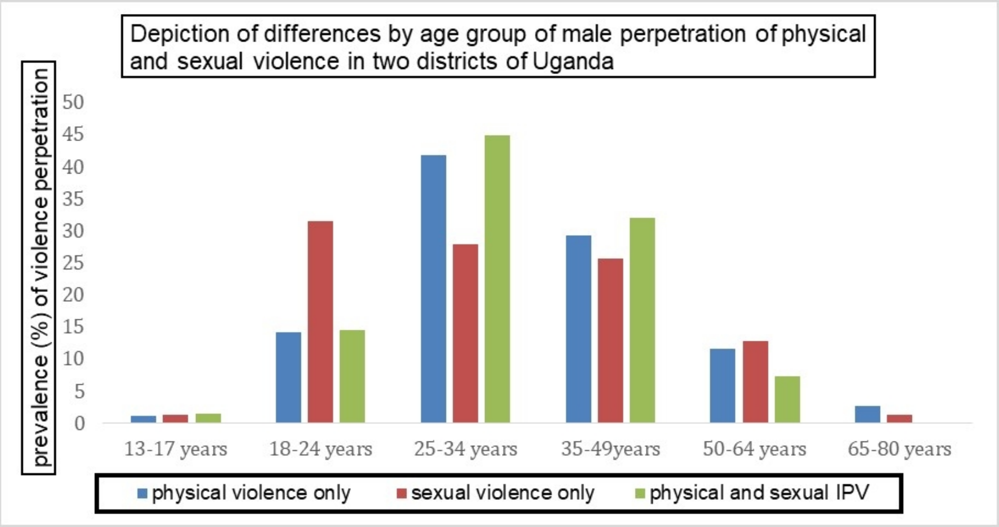

154x81mm (150 x 150 DPI)

Online supplementary Appendix1:

Sensitivity analysis: Characteristics of the analysed sample (N=1314) and non-responders (N=321)

Variable	Analysed sample N (%)	Non-responders N (%)	p-value
**Study Strata**			
Wakiso communities	**578**	**179**	0.364
**Urban -Kazo**	206(35.6)	68(37.9)	
**Semi-urban -Lukwanga**	219(37.9)	62(34.6)	
**Rural -Sentema**	153(26.5)	49 (27.4)	
Hoima communities	**736**	**142**	0.210
**Urban –Ishaka_Kijungu**	243(33.0)	67(47.2)	
**Semi-urban-Butem**	198(25.5)	39(27.5)	
**Rural -Kitoba**	295(40.1)	42 (29.6)	
**Age (mean, SD), interquartile range(years)**	34 ±12.9, 24-42	15.8 ±3.4, 14-17	<0.05
**Education**			
**Primary school and below**	688(52.3)	149(46.4)	>0.001
**Secondary school**	477(36.3)	111(34.5)	
**Post-secondary**	149(11.3)	61(19.0)	
**Not married**	151(11.5)	0	
**Religion**			
**Christianity**	1068(81.3)	230(71.7)	0.02
**Non-Christians**	246(18.7)	91(28.3)	
**HIV status from blood sample**			
**Negative**	1224(94.3)	310(96.6)	<0.001
**Positive**	74(5.7)	11(3.4)	

ew only

Online supplementary appendix 2

Questions used to assess food insecurity

Question	Responses
**Q.1 In the past 12 months, were there months in which you did not have enough food to meet your family's needs?**	Yes/ No
**Q.2 In the last 12 months, have you or other adults in your household withheld a meal because there was not enough food?**	Yes/ No
**Q.3 In the last 12 months, did you or other adults in your household ever not eat for a whole day because there wasn't enough food?**	Yes/ No
**Q. 4 In the last 12 months, did you ever *cut the size of any of the children's meals* because there wasn't enough food?**	Yes/No
**Q. 5 In the last 12 months how often were you worried that food would run out?**	Always/Sometimes/Never
**Q. 6 If yes in Q.5, How often did this happen?**	Almost every month/ sometimes but not every month/ only 1 or 2 months/Don't remember

For peer review only - http://bmjopen.bmj.com/site/about/guidelines.xhtml

STROBE Statement—Checklist of items that should be included in reports of ***cross-sectional studies***

	Item No	Recommendation	Page No
**Title and abstract**	1	(*a*) Indicate the study's design with a commonly used term in the title or the abstract	✓ Page1-2
		(*b*) Provide in the abstract an informative and balanced summary of what was done and what was found	✓ Page 2
**Introduction**			
Background/rationale	2	Explain the scientific background and rationale for the investigation being reported	✓ Page 4-6
Objectives	3	State specific objectives, including any prespecified hypotheses	✓ Page6
**Methods**			
Study design	4	Present key elements of study design early in the paper	✓ Page6-7
Setting	5	Describe the setting, locations, and relevant dates, including periods of recruitment, exposure, follow-up, and data collection	✓ Page7
Participants	6	(*a*) Give the eligibility criteria, and the sources and methods of selection of participants	✓ Page7-8
Variables	7	Clearly define all outcomes, exposures, predictors, potential confounders, and effect modifiers. Give diagnostic criteria, if applicable	✓ Page8-10
Data sources/ measurement	8*	For each variable of interest, give sources of data and details of methods of assessment (measurement). Describe comparability of assessment methods if there is more than one group	✓ *Page9-10*
Bias	9	Describe any efforts to address potential sources of bias	✓ Page7-8
Study size	10	Explain how the study size was arrived at	✓ Page7
Quantitative variables	11	Explain how quantitative variables were handled in the analyses. If applicable, describe which groupings were chosen and why	✓ Page 9-11
Statistical methods	12	(*a*) Describe all statistical methods, including those used to control for confounding	✓ Page 11
		(*b*) Describe any methods used to examine subgroups and interactions	x
		(*c*) Explain how missing data were addressed	x
		(*d*) If applicable, describe analytical methods taking account of sampling strategy	N/A
		(*e*) Describe any sensitivity analyses	✓ Page8
**Results**			
Participants	13*	(a) Report numbers of individuals at each stage of study—eg numbers potentially eligible, examined for eligibility, confirmed eligible, included in the study, completing follow-up, and analysed	✓ Page8
		(b) Give reasons for non-participation at each stage	x
		(c) Consider use of a flow diagram	x

For peer review only - http://bmjopen.bmj.com/site/about/guidelines.xhtml

Descriptive data	14*	(a) Give characteristics of study participants (eg demographic, clinical, social) and information on exposures and potential confounders	✓ Page13
		(b) Indicate number of participants with missing data for each variable of interest	NA
Outcome data	15*	Report numbers of outcome events or summary measures	✓ Page14
Main results	16	(*a*) Give unadjusted estimates and, if applicable, confounder-adjusted estimates and their precision (eg, 95% confidence interval). Make clear which confounders were adjusted for and why they were included	✓ Page15-16
		(*b*) Report category boundaries when continuous variables were categorized	NA
		(*c*) If relevant, consider translating estimates of relative risk into absolute risk for a meaningful time period	NA
Other analyses	17	Report other analyses done—eg analyses of subgroups and interactions, and sensitivity analyses	x
**Discussion**			
Key results	18	Summarise key results with reference to study objectives	✓ Page18
Limitations	19	Discuss limitations of the study, taking into account sources of potential bias or imprecision. Discuss both direction and magnitude of any potential bias	✓ Page20-21
Interpretation	20	Give a cautious overall interpretation of results considering objectives, limitations, multiplicity of analyses, results from similar studies, and other relevant evidence	✓ Page18-20
Generalizability	21	Discuss the generalizability (external validity) of the study results	✓ Page20
**Other information**			
Funding	22	Give the source of funding and the role of the funders for the present study and, if applicable, for the original study on which the present article is based	✓ Page22

*Give information separately for exposed and unexposed groups.

Note: An Explanation and Elaboration article discusses each checklist item and gives methodological background and published examples of transparent reporting. The STROBE checklist is best used in conjunction with this article (freely available on the Web sites of PLoS Medicine at http://www.plosmedicine.org/, Annals of Internal Medicine at http://www.annals.org/, and Epidemiology at http://www.epidem.com/). Information on the STROBE Initiative is available at www.strobe-statement.org.

For peer review only

BMJ Open

Household food insecurity and its association with self-reported male perpetration of intimate partner violence: a survey of two districts in Central and Western Uganda

Journal:	*BMJ Open*
Manuscript ID	bmjopen-2020-045427.R1
Article Type:	Original research
Date Submitted by the Author:	02-Feb-2021
Complete List of Authors:	Awungafac, George; Karolinska Institute, Department of Global Public Health<br>Mugamba, Stephen; Africa Medical and Behavioural Science Organization<br>Nalugoda, Fred; Africa Medical and Behavioural Science Organization<br>Sjöland, Carl Fredrik; Karolinska Institute, Department of Global Public Health<br>Kigozi, Godfrey; Africa Medical and Behavioural Science Organization<br>Rautiainen, S; Karolinska Institute, Department of Global Public Health<br>Malyabe, Robert; Africa Medical and Behavioural Science Organization<br>Ziegel, Leo; Karolinska Institute, Department of Global Public Health<br>Nakigozi, Gertrude; Africa Medical and Behavioural Science Organization<br>Nalwoga, Grace; Africa Medical and Behavioural Science Organization<br>Kyasanku, Emmanuel; Africa Medical and Behavioural Science Organization<br>Nkale, James; Africa Medical and Behavioural Science Organization<br>Watya, Stephen; Africa Medical and Behavioural Science Organization<br>Ekström, Anna; Karolinska Institute, Department of Global Public Health<br>Kågesten, Anna; Karolinska Institute, Department of Global Public Health
**Primary Subject Heading**:	Global health
Secondary Subject Heading:	Epidemiology, Public health, Health policy, HIV/AIDS
Keywords:	Public health < INFECTIOUS DISEASES, Epidemiology < INFECTIOUS DISEASES, HIV & AIDS < INFECTIOUS DISEASES, MENTAL HEALTH

SCHOLARONE™
Manuscripts

I, the Submitting Author has the right to grant and does grant on behalf of all authors of the Work (as defined in the below author licence), an exclusive licence and/or a non-exclusive licence for contributions from authors who are: i) UK Crown employees; ii) where BMJ has agreed a CC-BY licence shall apply, and/or iii) in accordance with the terms applicable for US Federal Government officers or employees acting as part of their official duties; on a worldwide, perpetual, irrevocable, royalty-free basis to BMJ Publishing Group Ltd ("BMJ") its licensees and where the relevant Journal is co-owned by BMJ to the co-owners of the Journal, to publish the Work in this journal and any other BMJ products and to exploit all rights, as set out in our licence.

The Submitting Author accepts and understands that any supply made under these terms is made by BMJ to the Submitting Author unless you are acting as an employee on behalf of your employer or a postgraduate student of an affiliated institution which is paying any applicable article publishing charge ("APC") for Open Access articles. Where the Submitting Author wishes to make the Work available on an Open Access basis (and intends to pay the relevant APC), the terms of reuse of such Open Access shall be governed by a Creative Commons licence – details of these licences and which Creative Commons licence will apply to this Work are set out in our licence referred to above.

Other than as permitted in any relevant BMJ Author's Self Archiving Policies, I confirm this Work has not been accepted for publication elsewhere, is not being considered for publication elsewhere and does not duplicate material already published. I confirm all authors consent to publication of this Work and authorise the granting of this licence.

For peer review only

Household food insecurity and its association with self-reported male perpetration of intimate partner violence: a survey of two districts in Central and Western Uganda

George Awungafac[1*], Stephen Mugamba[2], Fred Nalugoda[2], Carl Fredrik Sjöland[1], Godfrey Kigozi[2], Rautiainen, S[1], Robert Bulamba Malyabe[2], Leo Ziegel[1], Gertrude Nakigozi[1], Grace Kigozi Nalwoga[2], Emmanuel Kyasanku[2], James Nkale[2], Stephen Watya[2], Anna Mia Ekström[1], Anna Kågesten[1]

Author affiliations

1. Department of Global Public Health, Karolinska Institutet, Stockholm Sweden

2. Africa Medical and Behavioural Sciences Organisation, Kampala, Uganda

*Corresponding author: **awungafacg@yahoo.com**

peer review only

Abstract

Objectives: This study aimed to determine the lifetime prevalence of male perpetrated of intimate partner violence (IPV), and to assess its association with food insecurity, sociodemographic factors, and health-risk behaviours in Uganda, in the year preceding COVID-19 pandemic-associated lockdowns.

Design: Population-based household survey

Setting: Urban, semi-urban and rural communities of Wakiso and Hoima Districts of Uganda

Participants: The total survey sample of 2014 participants comprised of males aged 13-80 years. This study included males who reported having ever been in a sexual union and who responded to the IPV questions (N=1314).

Measures: Data was collected face-to-face using a questionnaire from May 2018 to July 2019. Lifetime IPV perpetration was measured as "no physical and/or sexual IPV", "physical" versus "sexual violence only", and both "physical and sexual violence". Past-year food insecurity was measured using questions from the Food Insecurity Experience Scale, and categorized into "none", "low" and "high". Multinomial logistic regression was used to determine the crude and adjusted relative risk ratios (aRRR) of IPV perpetration in relation to self-reported food insecurity, adjusting for sociodemographic and behavioural covariates.

Results: The prevalence of self-reported lifetime IPV was 14.6% and 6.5% for physical and sexual violence only, respectively. 5.3% of males admitted to perpetrating both physical and sexual IPV. Most (75.7%) males reported no food insecurity, followed by low (20.7%) and high (3.6%) food insecurity. In adjusted models, food insecurity was associated with increased risk of having perpetrated both physical and sexual violence (aRRR=2.57, 95%CI; 1.52-4.32). Number of sexual partners and alcohol consumption were associated with IPV perpetration, but not education level or religion.

For peer review only - http://bmjopen.bmj.com/site/about/guidelines.xhtml

Conclusion: Our results suggest that food insecurity drives male IPV perpetration; this could worsen with the SARS-CoV-2 outbreak.

Key words: Intimate Partner Violence, Men, Food insecurity, Uganda, HIV

Strengths and limitations of the study

- This is the first study to investigate the association between lifetime male perpetration of intimate partner violence with food insecurity in Uganda. We included a population-based, diverse and representative sample of participants from different geographic areas (rural, semi-urban and urban) in Uganda.
- The cross-sectional nature of the study precludes any conclusion about the temporal association between IPV and food insecurity.
- Gender-based violence including IPV is a sensitive topic, and may be subject to under-reporting and social desirability bias.

For peer review only

Introduction

Intimate partner violence (IPV) is a major global health problem and a human rights violation. IPV consists of intentional and abusive attitudes in the form of physical, sexual and/or emotional offense and controlling behaviours within an intimate partnership,(1). While IPV can take different forms, male perpetrated IPV is the most common form of violence against women (VAW),(2). Globally, one in three ever-partnered women report experiencing some form of physical and/or sexual violence in their lifetime, with the World Health Organization's Region for Africa (WHO AFRO) recording the highest prevalence (37%), followed by the WHO Region for the Americas (29.8%),(3). The WHO multi-country study on VAW estimated that the prevalence of male IPV perpetration was 21.1% for physical violence only, 11.7% for sexual violence only, and 11.8% for both physical and sexual violence,(4). Reports from diverse social and cultural contexts have demonstrated that IPV perpetration begins early in the life course, with most perpetrators reporting that they first engage in it during adolescence,(5).

IPV carries multiple, well-established consequences for women's health and wellbeing, with femicide being the most extreme form,(6,7). IPV is associated with increased risk of HIV transmission or of sexually transmitted infections (STI),(8,9) , severe depressive symptoms, as well as substance use and abuse, such as binge drinking,(10). Furthermore, children born to women experiencing IPV are at higher risk of premature death, poor health outcomes, and emotional and behavioural problems later in life,(11). Experiencing IPV has been shown to affect women and girls' social and economic empowerment,(12) , including decreased productivity at work, loss of employment opportunities and other important social engagement,(13).

Uganda has one of the highest burdens of IPV in the world. As such, women's experiences of IPV have been the main focus of research on VAW in the country. The 2014 Ugandan

For peer review only - http://bmjopen.bmj.com/site/about/guidelines.xhtml

Demographic and Health Survey (UDHS) found that 47% of married women had experienced physical IPV in their lifetime, with 29% reporting lifetime sexual IPV,(14). Slapping, hitting and beating pregnant women was found to be common, and has been shown to worsen during the course of pregnancy in Northern Uganda,(15). Furthermore, the country's HIV incidence has been found to correlate with the frequency and duration of exposure to IPV,(16).

While multiple intersecting factors drive male perpetration of VAW, there is growing evidence linking IPV with food insecurity,(17,18) which is defined as "a household-level economic and social condition of limited or uncertain access to adequate food",(19). For example, a cross-sectional study in slums of Kampala found that extreme physical violence perpetration and experience of such violence was common among a convenience sample of young men,(14-24 years), and was found to correlate with reported hunger and alcohol consumption, illicit drug use, poor mental health status and parental neglect due to alcohol,(20). In a report from South Africa by Gibbs et al., economic indicators of food insecurity ,such as unemployment and low earnings in the past month, were associated with IPV perpetration by men,(21). A small scale study from Abidjan, Cote d'Ivoire revealed that women with severe forms of food insecurity were at higher risk of experiencing IPV,(22). While studies from other regions, such as Nepal or the Unites States have established a significant relationship between food insecurity and both IPV perpetration and experiences among women, few studies from sub-Saharan Africa exist on this topic,(23,24). According to the United Nations (UN), food insecurity is increasing in Uganda, rising from 24.1% in 2006 to 41% in 2018,(25). The ongoing SARS-CoV-2 crisis has contributed to this considerable increase in Ugandan food insecurity and, in line with predictions of the World Bank and the World Food Programme, is likely to continue to do so,(26,27).

Male IPV perpetration against women is a complex, multi-level, social, economic and structural problem; one rooted in unequal gender norms and systems of power. In most cultural settings, a

For peer review only - http://bmjopen.bmj.com/site/about/guidelines.xhtml

gendered order exists that favours stereotypically masculine men's dominance over women, as well as over other more marginalised masculinities that do not live up to the norms of being a "real" man,(28). IPV can thus be viewed both as a symptom of, and a tool to achieve, this type of "hegemonic" masculinity and power,(29). Being unable to provide food for one's self or household could be interpreted as failing to meet stereotypical masculine norms, thereby forcing men to use alternative strategies (such as violence) to demonstrate their manhood,(18). Mental health issues (depression or anxiety), which occur when concerns about food availability are accompanied by poor coping mechanisms such as alcohol consumption, can further lead to IPV, as demonstrated by Hatcher and colleagues in peri-urban South Africa,(18). Furthermore, the concept of "patriarchal risk",(30) can help to theoretically explain the cultural and societal dependence of women on male family members for food and protection. As a result of this phenomenon, boys may eat bigger portions than girls, resulting in males being prioritized to eat when there is not enough food.

Given the increasing reports of food insecurity in Uganda, and the lack of pre-existing literature in low-and-middle income sub-Saharan African settings, it is important to investigate the possible links between food insecurity and male perpetrated IPV in the country. The present study aims to determine the prevalence of lifetime male IPV perpetration among a representative sample of males in two Ugandan districts, and to assess its association with food insecurity, social-demographics and health-risk behaviours. The evidence gained through this study will inform future programs and policies to prevent and better respond to IPV in Uganda and other similar settings.

For peer review only - http://bmjopen.bmj.com/site/about/guidelines.xhtml

Methods

Study design

Data were collected between May 2018 and July 2019, as part of a cross-sectional baseline survey of a longitudinal open population-level cohort established by the Africa Medical and Behavioural Sciences Organization (AMBSO) to conduct population health surveillance (PHS) in the Wakiso and Hoima districts of Uganda. In brief, the AMBSO PHS aims to generate evidence-based data in order to inform policy on the health status of the population through periodic monitoring of disease trends and determinants of health. The cohort includes males and females aged 13-80 years in the study communities, and collects yearly data on sociodemographic factors, violence including IPV, non-communicable diseases (diabetes, hypertension, cancers, etc.), communicable diseases (such as HIV and STIs), food and nutrition, immunization of children, health risks (alcohol use, illicit drug consumption, number of sexual partners, and circumcision) and mental health. The current study uses the baseline data with focus on men's lifetime perpetration of IPV in the study communities.

Study communities

With a population of nearly 2 million, Wakiso district lies in the Central Region of Uganda and surrounds parts of the capital, Kampala. Located in the Bunyoro Region of Mid-Western Uganda, Hoima district has a smaller population of roughly 570,000 individuals. The selection of these two districts was purposeful, based on their common and diverse characteristics. Wakiso has a high migrant worker population and is known to be a hotspot for sex workers and men who have sex with men. The discovery of oil in Hoima and increasing mining activities attract migrant workers, sex workers and fisher folks – populations known to have high risk of HIV,(31). Each district was stratified into urban, semi-urban and rural areas. Residents in all urban and semi-urban areas,

For peer review only - http://bmjopen.bmj.com/site/about/guidelines.xhtml

and those in a randomly selected rural community (representing the rural sub-counties), were included in the baseline survey.

Study population and sampling

The study population comprised all males aged 13-80 years in the study communities. A lower age limit of 13 years was considered because of the high prevalence of early sexual debut in Uganda,(32). This study sample was defined as males who reported ever having a sexual relationship. In each study community, the sampling frame included all households. In each household, all males aged 13-80 years were considered potential participants. A minimum sample of N=738 was needed to estimate the associations in the current study with 80% power at the alpha=0.05 level. To be included in the study, participants had to be able and willing to respond to the survey questions and to provide informed consent. The full survey sample consisted of 2014 male participants who were recruited into the AMBSO PHS cohort. Males who reported not ever having been in a sexual union (N=379) and those who abstained from responding to questions on male IPV perpetration (N=321) were excluded (Figure 1).

Even though the individual response rate was 80.4%, the final analysis sample (N=1314) was near double the estimated sample of N=738 participants needed to estimate the association. This 80.4% response rate is similar to that of a multi-country survey on male IPV perpetration in Asia,(4). A sensitivity analysis comparing the characteristics of those who responded to the IPV questions and those who did not respond is included in online supplementary appendix 1. The sensitivity analysis revealed that, in comparison with those who responded to the IPV questions, non-responders were more likely to be young, Christian and of lower educational level. There was no significant difference in food insecurity among non-responders and responders. Any observed differences did not affect the findings, as the final sample was more than double the minimum estimated sample needed to establish an association.

For peer review only

For peer review only - http://bmjopen.bmj.com/site/about/guidelines.xhtml

Procedures

Data was collected in the baseline using a structured questionnaire covering ten domains, including: sociodemographics, first sex experience, family planning practice, marital status and practices, food supply and food security, IPV, HIV testing services (HTS), HIV care and treatment services, prostate cancer and behaviour risk. Trained data collectors of the same sex as participants collected the data via face-to-face interviews, using tools translated into local languages (Luganda in Wakiso and Runyoro in Hoima).

In addition to these survey questions, the PHS collected 10 millilitres of venous blood samples that were used to determine the HIV and syphilis status of participants, and aliquots stored for other future studies. HIV status was determined following the Ugandan Ministry of Health (MOH) rapid test algorithm,(33) , and the rapid plasma regain serological (RPR) test was used to determine syphilis status of the participants.

Dependent variables

The dependent variable was adapted from the WHO multi-country study on VAW,(3) , and defined as lifetime self-reported perpetration of physical and/or sexual violence against an intimate (sexual) partner. Following the approach used by Fulu et al,(4) , the outcome was grouped into four independent and unordered categories: 1) no perpetration of physical and/or sexual violence (no IPV); 2) physical IPV perpetration only; 3) sexual IPV perpetration only; and 4) both physical and sexual IPV perpetration (Table 1). The “no IPV perpetration” category was used as the reference group, referring to males reporting to never have perpetrated physical and/or sexual violence against their intimate sexual partner. The “physical violence only” category consisted of males reporting to have perpetrated one or more forms of physical violence, but who had never used any sexual violence; “sexual violence only” was coded using the same approach but referring

For peer review only - http://bmjopen.bmj.com/site/about/guidelines.xhtml

to one or more forms of sexual violence. Males who reported to having perpetrated both physical and sexual violence in their lifetime were grouped into the "physical and sexual violence" category. Lifetime estimates were used in this study in order to capture all self-reports of IPV perpetration.

Table 1. Definition and measurement of lifetime IPV perpetration (dependent variable)

Type of violence perpetration	Have you ever done any of the following to your sexual partner?
**Physical violence only (“yes” to ≥1 indicator)**	- Push, slap or hold partner down?<br>- Punch with fist or with something that could hurt partner?<br>- Kick or drag partner?<br>- Tried to strangle or burn partner?<br>- Attacked partner with a knife, gun, or other type of weapon?
**Sexual violence only (“yes” to ≥1 indicator)**	- Force her to perform sexual acts against partner’s will?<br>- Used threats to force her to have sex when she did not want to?<br>- Physically force her to have sex against her wish?
**Physical and sexual violence (“yes” to ≥1 indicators of both)**	- Perpetration of both physical violence and sexual violence
**No IPV perpetration**	- No perpetration of both physical violence and sexual violence

Independent variables

Food insecurity

The main independent variable was past-year food insecurity, assessed using an adapted version of the Food Insecurity Experience Scale (FIES) questions,(34) in which six selected items were used to evaluate the household food situation during the preceding 12 months (Table 2).

Table 2: Assessment questions and coding for the measurement and classification of food insecurity among in the study.

Question	Responses	Coding of responses
Q.1 In the past 12 months, were there months in which you did not have enough food to meet your family’s needs?	Yes/ No	Yes=1, No=0
Q.2 In the last 12 months, have you or other adults in your household withheld a meal because there was not enough food?	Yes/ No	Yes=1, No=0
Q.3 In the last 12 months, did you or other adults in your household ever not eat for a whole day because there wasn’t enough food?	Yes/ No	Yes=1, No=0

For peer review only - http://bmjopen.bmj.com/site/about/guidelines.xhtml

Q. 4 In the last 12 months, did you ever *cut the size of any of the children's meals* because there wasn't enough food?	Yes/No	Yes=1, No=0
Q. 5 In the last 12 months, how often were you worried that food would run out?	Always/Sometimes/Never	Always=1, Sometimes=1, Never=0
Q. 6 If yes in Q.5, How often did this happen?	Almost every month/ sometimes but not every month/ only 1 or 2 months/Don't remember	Almost every month=1, sometimes but not every month=1, only 1 or 2 months=0, don't remember=0

Source: Food Insecurity Experience Scale (FIES)

In line with international definitions,(35) , negative responses to assessment questions such as "no" "never" "only 1 or 2 months" were coded as "0" (referring to those who reported having minor access problems for food during the preceding 12 months), while positive or affirmative responses such as "yes", "always", "almost every month", "some months but not every month" were coded as "1" (referring to having problems with access to adequate food during the preceding 12 months). All codes were summed to obtain a total score of up to six (i.e. 0-6) for food insecurity, with higher score indicating the extent of food insecurity. As adapted from international approaches(35), participants were categorised into the following categories: no food insecurity (0-1), low food insecurity (2-4) and high food insecurity (hunger) (5-6). Due to the small proportion reporting high food insecurity (3.6%), low food insecurity and high food insecurity were merged during the modelling procedures in line with previous studies,(18) , i.e. comparing food insecurity against food security in line with previous studies,(18).

Covariates

Sociodemographic covariates included study district (urban/semi-urban/rural); age; education level (categorized into primary school and below, secondary school and post-secondary), marital status (married or in a union/not married); religion (categorized into Christian, non-Christians); and having living children (yes/no). Health-risk behaviours included, number of lifetime sexual partners

For peer review only - http://bmjopen.bmj.com/site/about/guidelines.xhtml

(one partner/two or more partners), past-year condoms use (yes/no), use of illicit drugs (yes/no), alcohol consumption (yes/ no) and male circumcision (yes/no). The choice of covariates was based on previous studies indicating number of sexual partners as well as (low) condom use ,(36–38) and male circumcision,(38) ,as risk factors for IPV.

Statistical analysis

We first conducted descriptive analyses to explore the distribution of variables and to identify outliers. The analysis in this report were not weighted. Missing values for covariates were replaced using multiple imputation assuming random missingness. Pearson's Chi-squared test and Fishers exact test were used to compare proportions with α set at 5%. Next, we performed bivariate multinomial logistic regression (MLR) to determine the crude relative risk ratios (RRR) and 95% confidence intervals (CI) of IPV perpetration in relation to the independent variable and covariates. We followed the approach used by Fulu et al,(4) and used "No IPV perpetration" as the base outcome against which all other outcomes were compared, assuming that the association of food insecurity is different for each typology of violence. This type of regression produces RRR, which are the exponentiated coefficients of the regression, and explain the relative effect of the independent variable on the outcomes using one of the outcomes as a base outcome. A full model was built to explain the associated factors to IPV perpetration in relation to both food insecurity, health-risk behaviours and sociodemographic background. The full model comprised of all of the study variables in bivariate analysis, irrespective of significance levels, that are theoretically known to be associated to IPV perpetration. The final model comparing associated factors to IPV perpetration was selected through backward elimination using a maximum likelihood ratio (LR) test. All analyses were conducted using STATA version 16 (StataCorp), while Microsoft Office Excel was used to design figures.

For peer review only - http://bmjopen.bmj.com/site/about/guidelines.xhtml

Ethical considerations

The AMBSO PHS cohort study received ethical approval from the Clarke International University Ethics Committee (Ref:CIUREC/0059) as the local Institutional Review Board (IRB) of record, and clearance from the Ugandan Council of Science and Technology (Ref:SS4468). Clearance was obtained from both districts to conduct the study. All participants provided a written informed consent /assent before participating in the study. With the exception of emancipated minors (under 18 years) who (like adults) gave their own written or oral assent in addition to written, parental/guardians consent was obtained. Data was collected by a study team trained and certified in Human subject protection and Good clinical Practice. Interviews were conducted in private. Unique identification codes were used in all computerized data, and the lists that linked participants' names to the codes were accessible only to senior project staffs in password protected computers. Participants who tested positive for HIV infection and syphilis were linked to specialized care while those who needed other health support services were referred to health facilities of their choice.

Patients and Public Involvement statement

The study team worked hand in hand with community health workers, local community leaders and a community-research advisory structure to develop and clarify a common understanding of the aims of the population-based survey, the design and operational aspects as well as how results will be disseminated. As part of this "patient and public involvement" effort, a consensus was reached with the advisory committee on a plan to increase general public awareness of the study and health issues that were deemed areas of PHS focus.

For peer review only - http://bmjopen.bmj.com/site/about/guidelines.xhtml

Results

Sample characteristics

Table 3 shows the characteristics of the study participants. The mean age of respondents was 34 years (SD; ±12.9). Food insecurity was found to affect one in four men, with 20.7% and 3.6% categorized as low versus high food insecurity, respectively. About one third and 50% of men reported having had multiple sex partners and used alcohol in the past year respectively; and approximately 6% tested positive for HIV.

Table 3. Sample characteristics of male respondents in two districts of Uganda (N=1314)

Variable	n	%
Age (mean, SD)	34 ±12.9	
Education		
Primary and below	688	52.4
Secondary school	477	36.3
Post-secondary	149	11.3
Marital status		
Married or in a union	1163	88.5
Not married	151	11.5
Have any living children		
Yes	999	76.2
No	312	23.8
Religion		
Christianity	1068	81.3
Non-Christians	246	18.7
Past-year food security		
No food insecurity	995	75.7
Low food insecurity	272	20.7
High food insecurity	47	3.6
Score (mean, SD)	1.48 ±1.05	
Number of lifetime sexual partners		
One partner	919	69.9
Two or more partners	395	30.1
Current use of condoms		
Yes	519	39.5
No	795	60.5
HIV status from blood sample		
Negative	1224	94.3
Positive	74	5.7
Past-year alcohol use		
Yes	677	48.5

For peer review only - http://bmjopen.bmj.com/site/about/guidelines.xhtml

No	640	51.5
Circumcised		
Yes	659	50.2
No	655	49.8
Past-year use of illicit drugs		
Yes	43	3.3
No	1271	96.7

Prevalence of lifetime IPV perpetration

Table 4 presents the prevalence of perpetration of different forms of IPV against an intimate (sexual) partner by male participants. Most (73.6%) males reported never having perpetrated any form of IPV. The prevalence of self-reported physical IPV ranged from 19.3% (pushed, slapped, held down) to 0.9% (attacked partner with a knife, gun or other weapon, with 14.6% having perpetrated any form of physical violence only. Lifetime perpetration of sexual IPV ranged from 8.2% (threatened or pressured partner into sex when unwanted) to 5.4% (physically forced partner to have sex) and 2.4% (used other types of force to have sex), with 6.5% of males reporting having perpetrated any form of sexual IPV only. In total, 5.3% of males reported previous perpetration of both physical and sexual IPV.

Table 4: Prevalence of self-reported lifetime perpetration of IPV against a sexual partner among ever-partnered males aged 13-80 years in two districts of Uganda, May 2018 to July 2019

	**Lifetime IPV perpetration (N=1314)**	
	n	%
**Physical violence (any form)**	261	19.9
Push, slapped or held down partner	253	19.3
Kicked or dragged partner	54	4.1
Tried to strangle partner	19	1.5
Attacked partner with a knife, gun or other weapon	12	0.9
Punch with fist or with something that could hurt partner?	61	4.6
**Sexual violence (any form)**	155	11.8
Threatened or pressured partner into sex when unwanted	108	8.2
Physically forced partner into sex	70	5.4
Used other ways to force partner to perform sexual acts when unwanted	31	2.4
**Any physical or sexual violence**	347	26.4
**Physical IPV perpetration only**	192	14.6

For peer review only - http://bmjopen.bmj.com/site/about/guidelines.xhtml

**Sexual IPV perpetration only**	86	6.5
**Both physical and sexual IPV perpetration**	69	5.3
**No IPV perpetration**	967	73.6

Figure 2 shows the prevalence of self-reported IPV perpetration across different age groups. As can be seen, the prevalence of physical violence only and both physical and sexual violence was highest among 25-34-year-olds compared to the other age-groups ($p<0.001$), with perpetration of sexual violence only being most commonly reported by young males (18-24 years) and less common after age 34 ($p=0.517$).

Factors associated with IPV perpetration

Bivariate analysis

In bivariate analysis, food insecure participants had an RRR of 2.71 (95% CI 1.64-4.46) to have perpetrated both physical and sexual violence, but no significantly higher risk to have perpetrated physical vs. sexual violence only, respectively (Table 5). Compared to the youngest age group 13-24 years, young adult participants' (25-34 years) had a RRR of 2.59 (95%CI 1.64-4.07) for perpetrating physical violence only, and 2.64 (95%CI 1.30-5.36) for both physical and sexual violence, and the RRR for those aged 35+ years was 2.16 (95%CI 1.38-3.38) for physical violence. Being married was associated with a lower relative risk of having perpetrated sexual violence only. Having living children and reporting no condom use was associated with perpetration of physical violence only. Education level, was not significantly associated with IPV perpetration. Reported use of illicit drugs during the preceding year was associated with perpetration of physical violence only, and both physical and sexual violence.

For peer review only - http://bmjopen.bmj.com/site/about/guidelines.xhtml

Table 5: Crude Relative Risk Ratios (RRR) of male IPV perpetration in relation to food insecurity and covariates in two districts of Uganda (baseline outcome=no IPV) (N=1314) 2019

Variable	N	No IPV	Physical violence only			Sexual violence only			Both Physical and sexual violence		
		N (%)*	N (%)*	RRR, 95% CI	p-value	N (%)*	RRR, 95% CI,	p-value	N (%)*	RRR, 95% CI,	p-value
Age group(years)											
13-24 (Ref)	353	285(80.7)	29(8.2)			28(7.9)			11(3.1)		
25-34	439	304(69.3)	80(18.2)	2.59(1.64-4.07)	0.001	24(5.5)	0.80(0.46-1.42)	0.451	31(7.1)	2.64(1.30-5.36)	0.007
35+	522	378(72.4)	83(15.9))	2.16(1.38-3.38)	0.001	34(6.5)	0.92(0.54-1.54)	0.741	27(5.2)	1.85(0.90-3.79)	0.093
Education											
Primary or below(Ref)	688	499(72.5)	106(15.4)			49(6.7)			37(5.4)		
Secondary school	477	349(73.2	68(14.3)	0.92(0.66-1.28)	0.612	34(7.1)	1.05(0.66-1.68)	0.815	26(5.5)	1.0(0.60-1.69)	0.986
Post-secondary	149	119(79.9)	18(12.1)	0.71(0.42-1.21)	0.216	6(4.0)	0.55(0.23-1.31)	0.176	6(4.0)	0.68(0.28-1.65)	0.393
Religion											
Islam and others (Ref)	242	186(76.9)	24(9.9)			16(6.6)			16(6.6)		
Christian religion	1072	781(72.8)	168(15.7)	1.66(1.06-2.63)	0.028	70(6.5)	1.04(0.59-1.83)	0.887	53(4.9)	0.79(0.44-1.41)	0.424
Marital status											
Not married (Ref)	151	109(72.2)	15(9.9)			18(11.9)			9(5.9)		
Married	1163	858(73.8)	177(15.2)	1.50(0.85-2.63)	0.159	68(5.9)	0.48(0.27-0.84)	0.010	60(5.2)	0.85(0.41-1.75)	0.655
Have living children											
No (Ref)	312	258(82.7)	18(5.7)			25(8.0)			11(3.5)		
Yes	999	708(70.9)	172(17.2)	3.48(2.10-5.77)	<0.001	61(6.1)	0.89(0.54-1.45)	0.636	58(5.8)	1.92(0.99-3.71)	0.053
Past-year Alcohol consumption											
No (Ref)	637	511(80.2)	74(11.6)			32(5.0)			20(3.1)		
Yes	677	456(67.4)	118(17.4)	1.79(1.30-2.45)	<0.001	54(7.9)	1.89(1.20-1.98)	0.006	49(7.3)	2.74(1.61-4.68)	<0.001
Past year illicit drug											
No (Ref)	1271	948(74.6)	180(14.2)			83(6.5)			60(4.7)		
Yes	43	19(44.2)	12(27.9)	3.32(1.59-6.97)	0.001	3(6.9)	1.80(0.52-1.22)	0.351	9(20.9)	7.48(3.24-17.2)	<0.001

For peer review only - http://bmjopen.bmj.com/site/about/guidelines.xhtml

Variable	N	No IPV	Physical violence only			Sexual violence only			Both Physical and sexual violence		
Food insecure											
No food insecurity (Ref)	995	756(76.0)	139(14.1)			56(5.7)			31(3.2)		
Food insecure	319	214(67.1)	52(16.3)	1.31(0.92-1.86)	0.395	23(7.2)	1.28(0.77-1.22)	0.097	30(9.4)	2.71(1.64-4.46)	<0.001
Condom use in past 12 months											
No(Ref)	795	571(71.8)	130(16.4)			50(6.3)			44(5.5)		
Yes	519	396(76.3)	62(11.9)	0.69(0.49-0.96)	0.026	36(6.9)	1.04(0.66-1.62)	0.870	25(4.8)	0.82(0.49-1.36)	0.441
Circumcised											
No(Ref)	655	464(70.8)	107(16.3)			43(6.6)			41(6.3)		
Yes	659	503(76.3)	85(12.9)	0.73(0.54-1.00)	0.050	43(6.5)	0.92(0.59-1.43)	0.720	28(4.3)	0.63(0.38-1.03)	0.068
Number of lifetime sexual partner											
One (Ref)	919	791(76.3)	124(13.5)			55(6.0)			39(4.2)		
Two or more	395	266(67.3)	68(17.2)	1.44(1.04-2.01)	0.028	31(7.8)	1.48(0.94-2.36)	0.098	30(7.5)	2.03 (1.23-3.33)	0.005
Hoima district (Ref: Wakiso)	736	526(71.5)	112(15.2)	1.17(0.86-1.61)	0.912	55(7.5)	1.49(0.94-2.35)	0.679	43(5.8)	1.38 (0.84-2.29)	0.875

*Row percentage

For peer review only

For peer review only - http://bmjopen.bmj.com/site/about/guidelines.xhtml

Multivariable analysis

Table 6 illustrates the adjusted relative risk (aRRR) for IPV perpetration in relation to food insecurity, controlling for sociodemographic factors and health risk behaviours. Food insecurity remained significantly associated with self-reported perpetration of both physical and sexual violence (aRRR=2.57, 95%CI; 1.52-4.32). In terms of covariates, having two or more sexual partners also remained associated with perpetration of physical violence only, and both physical and sexual violence. Alcohol consumption remained associated with all typologies of IPV perpetration under study. Reported use of illicit drugs remained associated with physical violence and both physical and sexual violence. Having living children was associated with perpetration of physical only, while being married appeared to be protective against perpetration of sexual violence only. Age and religion were not associated with any IPV perpetration in the adjusted model.

Table 6: Adjusted relative risk ratios (aRRR) of male IPV perpetration in relation to food insecurity in two districts of Uganda, controlling for sociodemographics and health-risks behaviours (N=1314)

variable	Physical violence only*		Sexual violence only*		Both physical and sexual violence*	
	Crude RRR, 95% CI	aRRR, 95% CI	Crude RRR, 95% CI	aRRR, 95% CI	Crude RRR, 95% CI	aRRR, 95% CI
Food-insecurity (ref: no food insecurity)	1.31 (0.92-1.86	1.19 (0.82-1.73)	1.28 (0.77-2.12	1.33 (0.54-1.55)	2.71 (1.64-4.46)	2.57 (1.52-4.32)
Two or more sexual partners (ref: one partner)	1.0 (1.04-2.01)	1.64 (1.15-2.33)	1.48 (0.94-2.36)	1.38 (0.85-2.25)	2.03 (1.23-3.33)	1.97 (1.15-3.37)
Drink alcohol (ref: no)	1.79 (1.30-2.45)	1.51 (1.07-2.12)	1.89 (1.20-2.98)	1.89 (1.16-3.07)	2.74 (1.61-4.68)	2.55 (1.42-4.58)
Illicit drug use the past year (ref: no)	3.32 (1.59-6.97)	3.84 (1.73-8.52)	1.80 (0.52-6.22)	1.29 (0.36-4.58)	7.48 (3.24-17.2)	4.80 (1.93-11.9)
Sociodemographic covariates						
25-34 years (ref: 18-24 years)	2.59 (1.64-4.07)	1.37 (0.99-1.91)	0.80 (0.46-1.42)	0.84 (0.51-1.39)	2.64 (1.30-5.36)	1.66 (0.99-2.78)
35+ years	2.16(1.38-3.38)	0.95(0.53-1.68)	0.92(0.59-1.83)-	0.80(0.41-1.54)	0.79(0.44-1.41)	0.40(0.19-0.84)

For peer review only - http://bmjopen.bmj.com/site/about/guidelines.xhtml

Christian religion (ref: non-Christians)	1.66 (1.06-2.63)	1.53 (0.94-2.51)	1.04 (0.59-1.83)	0.82 (0.45-1.51)	0.79 (0.44-1.41)	0.61 (0.32-1.17)
Married (Ref: not married	1.50(0.85-2.63)	1.34 (0.76-2.44)	0.48(0.27-0.84)	0.49 (0.27-0.86)	0.85(0.41-1.75)	0.81 (0.37-1.75)
Have children (ref: no)	3.48 (2.10-5.77)	3.24 (1.91-5.52)	0.89 (0.54-1.45)	0.92 (0.55-1.56)	1.92 (0.99-3.71)	1.77 (0.87-3.61)

*Reference/base outcome: no IPV perpetration.

Discussion

For peer review only

The current study set out to explore the prevalence of lifetime male IPV perpetration in two Ugandan districts, and its association with food insecurity. We found that one in four men reported ever perpetrating any form of physical and/or sexual violence against their intimate partners, and 5.3% reported perpetrating both physical and sexual IPV. Food insecurity was associated with male perpetration of both physical and sexual violence.

The prevalence of lifetime perpetration of IPV reported by this study are lower than that of other studies. Obtaining accurate data on the true burden of VAW is a global concern, due to under reporting,(39). It is likely that operating an open cohort like the AMBSO raises community awareness about laws and litigations in place for VAW, and that, out of fear of consequences, people feel scared to initially provide truthful responses. In this first round of the assessments, it is possible that the communities have not yet built enough trust with survey teams to report such sensitive information. Comparing the prevalence estimates of this study with data from previous studies is difficult, mainly due to the existence of limited research in a sub-Saharan African context, and the non-standardized ways in which surveys assess male IPV perpetration. For instance, although the 2006 Uganda demographic and health survey (DHS),(14) found that 47% of males perpetrated IPV, this finding was solely based on reports of physical violence (e.g. partner hitting, slapping, and kicking), without taking into account the perpetration of sexual IPV. Another large-

For peer review only - http://bmjopen.bmj.com/site/about/guidelines.xhtml

scale study that focused on past-year perpetration (rather than lifetime) in the Rakai District of Uganda indicated a prevalence of 10.4% for physical violence and 3.1% for sexual violence,(38). In the present study, 19.3% of men reported ever slapping or pushing their intimate partners, an estimate that is similar to other countries including Ghana (17.4%),(40) and Vietnam (23.5%),(41) , but lower than those obtained by the International Men and Gender Equality study (IMAGES) in the Democratic Republic of Congo (45.1%) and Rwanda (39.1%),(42).

One in four men were found to be food insecure, and food insecurity was associated with almost three times the relative risk of having perpetrated both physical and sexual IPV. Our findings are consistent with results of a recent study from a peri-urban setting of South Africa, in which food insecurity doubled the odds of males' perpetration of IPV,(18). The findings from our study also support the results of studies from Nepal,(25) and USA,(24), which found that food insecurity was more common among married women who had experienced physical violence from their intimate sexual partners. In the present context of the SARS-CoV2 pandemic, food insecurity may worsen, leading to a considerably increase in the risk of male IPV perpetration. Previous research indicates that the association between food insecurity and IPV perpetration may be driven by mental health problems such as anxiety and depression that arise from concerns about food availability,(18). This association is also believed to be connected to the conservation of gender norms, whereby the inability of men to provide for their partners and households affects their perceived masculinity, and contributes to their use of violence,(43). For example, a qualitative study among women and men in Bangladesh (44) found that inadequate food portions offered to men may trigger retaliatory acts of violence against their wives, and that men could withhold resources used to acquire food as a form of power. In line with other studies, the present study found that, after adjusting for food insecurity and sociodemographics, men who had consumed alcohol in the past year and who have multiple sexual partners were at significantly higher risk of perpetrating IPV,(4,45,46). Although we identified an association between food insecurity and IPV perpetration, some previous

For peer review only - http://bmjopen.bmj.com/site/about/guidelines.xhtml

literature has not demonstrated a direct pathway between the two. Mediating factors examined through structural equation models include poor mental health, gender attitudes, controlling behaviours and alcohol consumption (18,21). In interpreting the association between food insecurity and IPV perpetration, factors such as alcohol and having multiple partners are shown to have direct effects on IPV perpetration,(21)). Other literature have also analysed alcohol consumption and having multiple partners as mediators of IPV perpetration,(18,21).

Furthermore, we further found the prevalence of IPV perpetration to vary by age group, with reports of sexual and physical IPV being more common among young adult males compared to older men. These findings are in line with previous studies from the US,(46,47). It is possible that a relative lack of relationship experiences among young men, and gender-related vulnerabilities (constructed by the social and political system) could increase the tendency to perpetrate violence,(48). Peer influence has also been linked to IPV perpetration in young people,(46,47). These findings have implications for the design of future preventive interventions for different sub-populations, especially men in the younger age groups.

Our findings highlight the need for early prevention of IPV that targets young men, and the necessity to investigate and respond to food insecurity within the framework of IPV prevention. This study also emphasizes the need to confront violence against women in a multi-faceted manner, as in the planning of existing interventions in Uganda such as the SASA community based program in Kampala,(49). The Program P initiative,(50) provides concrete guidelines to be implemented by IPV programs in efforts to engage men in maternal and child health activities, supporting active fatherhood from time of pregnancy till early childhood globally. The SASA community-based program and Program P initiative also contain comprehensive strategies (including HIV risk reduction), and should be strengthened and scaled up to other parts of the country. There is a need to adapt context-relevant interventions to address food insecurity,

For peer review only - http://bmjopen.bmj.com/site/about/guidelines.xhtml

especially during the current COVID-19 crisis where severe increase in both IPV and food insecurity have been reported world-wide. This is of particular importance in Sub-Saharan Africa, where a huge surge in poverty rates and food insecurity is expected,(51,52). Thus, the results of this study (which show an association between food insecurity and IPV) suggest that IPV may become an even larger public health issue in Uganda and beyond in the near future. Potential interventions could include: efforts to boost agricultural food production such as short-term provision of seeds and tools to rehabilitate farming and facilitating the acquisition of credits for small businesses, conditional cash transfers to women household heads to enable them purchase necessities and pay for health expenses; implementation of school fee waivers and school feeding programs, and the provision of food vouchers to enable purchase of food in local markets and the emergency distribution of food,(53). Structural interventions to address IPV should be advocated for, including strengthening of legislation, training and monitoring justice departments in IPV-related issues, putting in place systems to encourage women to report incidences of IPV and setting up support and counselling centres for women who experience IPV,(54).

Limitations

This study is one of the first to investigate lifetime perpetration of different forms of physical and sexual violence among both younger and older men, and to examine their association with food insecurity and male IPV perpetration in a population-based, representative cohort in rural, urban and semi-urban Uganda. The findings of this study add to the existing literature on VAW in Uganda by using men’s self-reports of perpetration. However, given the self-reported nature of the data on a sensitive topic like IPV, it is possible that under-reporting of physical and sexual IPV as well as recall bias occurred. Some participants may have considered the questions to be too private and/or shameful to talk about, some may also have feared legal implications (given that IPV is a legal offense), and some respondents may have found it challenging to respond honestly to

For peer review only - http://bmjopen.bmj.com/site/about/guidelines.xhtml

questions about IPV. Therefore, there is a possibility that the true rate of IPV perpetration may have been higher than reported. Despite this limitation, the training of data collectors may have improved the confidence in self-reporting IPV perpetration by males. Even though the minimum sample needed for this study was attained, a good number of men did not respond to the IPV questions in this baseline survey. The sensitivity analysis revealed, however, that this had no significant effect on the findings. Although we did not have the data necessary to analyse income levels or employment status of the men, it is likely that food insecurity is a reflection of income level and the availability and access to livelihood resources. Finally, due to the cross-sectional nature of the data, we were unable to establish a temporal association between food insecurity and IPV perpetration in males. Even though multivariate analysis was performed in this study, the likelihood of residual confounding still remains.

Conclusions

The prevalence of male IPV perpetration in these Ugandan communities varies by age group; the higher rates of violence among young adult men must be addressed in IPV preventive strategies and programming. In these communities, food insecurity should be considered an underlying determinant of intimate sexual partner VAW. Moving forward, IPV and health-risk reduction interventions must be integrated and strengthened through the employment of strategies that consider the possibility of food insecurity as the driver of violence in intimate relationships. Considering the possible impact of COVID-19 lockdowns, and multiple reports on increased IPV as well as expected huge increases in poverty rates and food insecurity throughout Uganda, the need for preventive interventions is even greater than before. Longitudinal surveys are needed to investigate the drivers of food insecurity in Uganda and other low-income countries , and to determine its impact on different forms of violence.

For peer review only - http://bmjopen.bmj.com/site/about/guidelines.xhtml

Contributorship statement

The original ideas leading to the Population Health Surveillance study from which this manuscript is derived, were put together by SM, FN, GK, GN, SW and AME. GA and AEK conceptualised the research questions for the current study. GA analysed the data, wrote the first draft of the study and oversaw the manuscript development process. SM, FN, SW and GK coordinated the participant recruitment as well as the data collection, management and entry. RB, LZ, GN, GKN, EK and JN participated in actual data collection from the study participants. CF and SRL contributed in describing the measurement and classification of food insecurity. AEK provided oversight to the study, providing edits, structure and scope to the study together with AME. All authors reviewed the drafts leading to this final manuscript.

Funding

This study was made possible by private funds from AMBSO directors, the Karolinska Institute through a Swedish Research Council grant (grant number:2019-04474), and the Swedish Institute (SI) which supported the first author with an educational study grant (Ref:02205/2019).

Acknowledgement

We thank the staff of Africa Medical and Behavioral Sciences Organization (AMBSO); the PHS study participants; the local community leadership; and the Wakiso and Hoima Districts Directorates of Health services for supporting this work.

For peer review only - http://bmjopen.bmj.com/site/about/guidelines.xhtml

Conflict of interest

None declared

Data availability statement

The data for this study is strictly not available to the public. A reasonable request for the data can be made by contacting Stephen Mugamba (smugamba@gmail.com)

References

1. Krug EG et al., eds. World report on violence and health. Geneva, World Health Organization, 2002.

2. Richardson JR, Feder G, Coid J. Domestic violence affects women more than men. BMJ Br Med J. 2002;325(7367):779.

3. World Health Organisation. Global and regional estimates of violence against women: prevalence and health effects of intimate partner violence and non-partner sexual violence. Geneva; 2013.

4. Fulu E, Jewkes R, Roselli T, Garcia-Moreno C. Prevalence of and factors associated with male perpetration of intimate partner violence: findings from the UN Multi-country Cross-sectional Study on Men and Violence in Asia and the Pacific. Lancet Glob Heal. 2013 Oct 1;1(4):e187–207.

5. Peitzmeier SM, H MSP, Kågesten A, H MP, Acharya R, Ph D, et al. Intimate Partner Violence Perpetration Among Adolescent Males in Disadvantaged Neighborhoods Globally. 2016;1–7.

6. Martin PM, Carvajal N. Feminicide as 'act' and 'process': a geography of gendered violence in Oaxaca. Gender, Place Cult. 2016;23(7):989–1002.

7. Prieto-Carrón M, Thomson M, Macdonald M. No more killings! Women respond to femicides in Central America. Gend Dev. 2007;15(1):25–40.

8. Campbell JC, Baty ML, Ghandour RM, Stockman JK, Francisco L, Wagman J. The intersection of intimate partner violence against women and HIV/AIDS: a review. Int J Inj Contr Saf Promot.

For peer review only - http://bmjopen.bmj.com/site/about/guidelines.xhtml

2008;15(4):221–31.

9. Coker AL. Does physical intimate partner violence affect sexual health?: A systematic review. Vol. 8, Trauma, Violence, and Abuse. Sage PublicationsSage CA: Los Angeles, CA; 2007. p. 149–77.

10. Tsai AC, Wolfe WR, Kumbakumba E, Kawuma A, Hunt PW, Martin JN, et al. Prospective Study of the Mental Health Consequences of Sexual Violence Among Women Living With HIV in Rural Uganda. J Interpers Violence. 2015;31(8):1531–53.

11. Hossain MA, Sumi NS, Haque ME, Bari W. Consequences of Intimate Partner Violence Against Women on Under-Five Child Mortality in Bangladesh. J Interpers Violence. 2014;29(8):1402–17.

12. The World Bank. The Cost of Violence. Washington DC; 2009.

13. López-Sánchez MJ, Belso-Martínez JA, Hervás-Oliver JL. A Review of Economic Consequences and Costs of Male Violence Against Women. Indian J Gend Stud. 2019;26(3):424–34.

14. Ogland EG, Xu X, Bartkowski JP, Ogland CP. Intimate Partner Violence Against Married Women in Uganda. J Fam Violence. 2014 Nov 18;29(8):869–79.

15. Clarke S, Richmond R, Black E, Fry H, Obol JH, Worth H, et al. Intimate partner violence in pregnancy : sectional study from post- - conflict a cross- - northern Uganda. 2019;1–9.

16. Kouyoumdjian FG, Calzavara LM, Bondy SJ, O'Campo P, Serwadda D, Nalugoda F, et al. Intimate partner violence is associated with incident HIV infection in women in Uganda. Aids. 2013;27(8):1331–8.

17. Food and Agricultural Organisation of the United Nation. How can food security interventions contribute to reducing gender-based violence ?Issue Brief. 2017;

18. Hatcher AM, Stöckl H, McBride R-S, Khumalo M, Christofides N. Pathways From Food Insecurity to Intimate Partner Violence Perpetration Among Peri-Urban Men in South Africa. Am J Prev Med. 2019 May 1;56(5):765–72.

19. Food Insecurity: A Public Health Issue. Public Health Rep. 2016;131(5):655.

20. Swahn MH, Gressard L, Palmier JB, Kasirye R, Lynch C, Yao H. Serious violence victimization and perpetration among youth living in the slums of Kampala, Uganda. West J Emerg Med. 2012;13(3):253–9.

For peer review only - http://bmjopen.bmj.com/site/about/guidelines.xhtml

21. Id AG, Jewkes R, Willan S, Washington L. Associations between poverty , mental health and substance use , gender power , and intimate partner violence amongst young (18- 30) women and men in urban informal settlements in South Africa : A cross-sectional study and structural equation model. 2018;(January 2017):1–19.

22. Fong S, Gupta J, Kpebo D, Falb K. Food insecurity associated with intimate partner violence among women in Abidjan , Cote d ' Ivoire An examination of femicides in Peru between 2009 and 2014. 2016;341–2.

23. Ricks JL, Cochran SD, Arah OA, Williams JK, Seeman TE. Food insecurity and intimate partner violence against women: Results from the California Women's Health Survey. Public Health Nutr. 2016;19(5):914–23.

24. Diamond-Smith N, Conroy AA, Tsai AC, Nekkanti M, Weiser SD. Food insecurity and intimate partner violence among married women in Nepal. J Glob Health. 2019 Jun;9(1):010412.

25. Uganda's food insecurity worsens - UN report [Internet]. [cited 2020 Apr 30]. Available from: https://www.independent.co.ug/ugandas-food-insecurity-worsens-un-report/

26. Programme WF. COVID-19 will double number of people facing food crises unless swift action is taken | World Food Programme [Internet]. 2020. Available from: https://www.wfp.org/news/covid-19-will-double-number-people-facing-food-crises-unless-swift-action-taken

27. The World Bank. Food Security and COVID-19 [Internet]. [cited 2020 May 15]. Available from: https://www.worldbank.org/en/topic/agriculture/brief/food-security-and-covid-19

28. Jewkes R, Morrell R. Hegemonic Masculinity , Violence , and Gender Equality : Using Latent Class Analysis to Investigate the Origins and Correlates of Differences between Men. 2018;21(4):547–71.

29. Jewkes R, Morrell R. Gender and sexuality : emerging perspectives from the heterosexual epidemic in South Africa and implications for HIV risk and prevention. 2010;1–11.

30. Cain M, Khanam SR, Nahar S. Class, Patriarchy, and Women's Work in Bangladesh. Popul Dev Rev. 1979 Sep;5(3):405.

31. Kamali A, Nsubuga RN, Ruzagira E, Bahemuka U, Asiki G, Price MA, et al. Heterogeneity of HIV incidence: a comparative analysis between fishing communities and in a neighbouring rural general population, Uganda, and implications for HIV control. Sex Transm Infect. 2016 Sep

For peer review only

For peer review only - http://bmjopen.bmj.com/site/about/guidelines.xhtml

1;92(6):447–54.

32. Uganda Bureau of Statistics (UBOS) and ICF. 2018. Uganda Demographic and Health Survey 2016. Kampala, Uganda and Rockville, Maryland, USA: UBOS and ICF.

33. Uganda. Ministry of Health. 2003. Uganda national policy guidelines for HIV voluntary counselling and testing. Kampala: Ministry of Health.

34. Ballard, T.J., Kepple, A.W. & Cafiero, C. 2013. The food insecurity experience scale: developing a global standard for monitoring hunger worldwide. Technical Paper. Rome, FAO. (available at http://www.fao.org/economic/ess/ess-fs/voices/en/).

35. Bickel, Gary; Nord, Mark; Price, Cristofer; Hamilton, William; Cook J. Guide to Measuring Household Food Security,; prepared by Bickel, Bickel Gary Mark, Nord Cristofer, Price William, Hamilton and John, Cook. 2000;82.

36. Roberts TA, Auinger P, Klein JD. Intimate partner abuse and the reproductive health of sexually active female adolescents. J Adolesc Heal. 2005;36(5):380–5.

37. Raj A, Santana MC, La Marche A, Amaro H, Cranston K, Silverman JG. Perpetration of intimate partner violence associated with sexual risk behaviors among young adult men. Am J Public Health. 2006;96(10):1873–8.

38. Mullinax M, Grilo SA, Song XS, Wagman J, Mathur S, Nalugoda F, et al. HIV-Risk Behaviors of Men Who Perpetrate Intimate Partner Violence in Rakai, Uganda. AIDS Educ Prev. 2017 Dec;29(6):527–39.

39. Palermo T, Bleck J, Peterman A. Tip of the iceberg: Reporting and gender-based violence in developing countries. Am J Epidemiol. 2014;179(5):602–12.

40. Chirwa ED, Sikweyiya Y, Addo-Lartey AA, Ogum Alangea D, Coker-Appiah D, Adanu RMK, et al. Prevalence and risk factors of physical or sexual intimate violence perpetration amongst men in four districts in the central region of Ghana: Baseline findings from a cluster randomised controlled trial. Dalby AR, editor. PLoS One. 2018 Mar 9;13(3):e0191663.

41. Yount KM, Higgins EM, VanderEnde KE, Krause KH, Minh TH, Schuler SR, et al. Men's Perpetration of Intimate Partner Violence in Vietnam. Men Masc. 2016 Apr 24;19(1):64–84.

42. Fleming PJ, Mccleary-sills J, Morton M, Levtov R, Heilman B, Barker G. Risk Factors for Men ' s

For peer review only

Lifetime Perpetration of Physical Violence against Intimate Partners : Results from the International Men and Gender Equality Survey (IMAGES) in Eight Countries. 2015;1–18.

43. Cardoso LF, Gupta J, Shuman S, Cole H, Kpebo D, Falb KL. What Factors Contribute to Intimate Partner Violence Against Women in Urban, Conflict-Affected Settings? Qualitative Findings from Abidjan, Côte d'Ivoire. J Urban Heal. 2016 Apr 21;93(2):364–78.

44. Lentz EC. Complicating narratives of women's food and nutrition insecurity: Domestic violence in rural Bangladesh. World Dev. 2018 Apr 1;104:271–80.

45. Culbreth R, Swahn MH, Salazar LF, Kasirye R, Musuya T. Intimate Partner Violence and Associated Risk Factors Among Youth in the Slums of Kampala. J Interpers Violence. 2019 Nov 29;088626051988992.

46. Swartout KM. The company they keep: How peer networks influence male sexual aggression. Psychol Violence. 2013;3(2):157–71.

47. Stark L, Seff I, Hoover A, Gordon R, Ligiero D, Massetti G. Sex and age effects in past-year experiences of violence amongst adolescents in five countries. PLoS One. 2019;14(7):1–13.

48. Mckool M, Stephenson R, Winskell K, Tharp T, Parrott D, Arbor A. Case for a Social Norms Approach. 2019;

49. Abramsky T, Devries K, Kiss L, Francisco L, Nakuti J, Musuya T, et al. A community mobilisation intervention to prevent violence against women and reduce HIV/AIDS risk in Kampala, Uganda (the SASA! Study): study protocol for a cluster randomised controlled trial. Trials. 2012;13:96.

50. Promundo, CulturaSalud, and REDMAS (2013). Program P – A Manual for Engaging Men in Father- hood, Caregiving, Maternal and Child Health. Promundo: Rio de Janeiro, Brazil and Washington, D.C. USA. These materials can be reproduced provided credit is given.

51. Lindberg LD et al., Early Impacts of the COVID-19 Pandemic: Findings from the 2020 Guttmacher Survey of Reproductive Health Experiences, New York: Guttmacher Institute, 2020, https://www.guttmacher.org/report/early-impacts-covid-19-pandemic-findings-2020-.

52. World Food Program. COVID-19 Level 3 Emergency: External Situation Report no.17. 2020.

53. Rose DD. Interventions to reduce household food insecurity: a synthesis of current concepts and

For peer review only - http://bmjopen.bmj.com/site/about/guidelines.xhtml

approaches for Latin America. Rev Nutr. 2008;21(suppl):159s-173s.

54. Bourey C, Williams W, Bernstein EE, Stephenson R. Systematic review of structural interventions for intimate partner violence in low- and middle-income countries: Organizing evidence for prevention Health behavior, health promotion and society. BMC Public Health. 2015;15(1).

Figure 1: Study flow, illustrating the selection process for inclusion into the analysis

Figure 2: Depiction of lifetime prevalence by age group of male lifetime IPV perpetration in two districts of Uganda, May 2018 to July 2019.

For peer review only

For peer review only - http://bmjopen.bmj.com/site/about/guidelines.xhtml

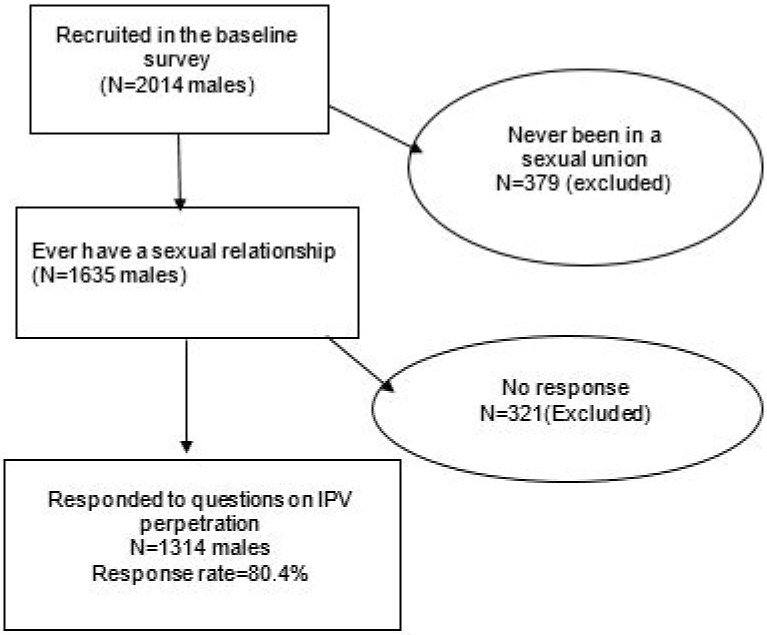

Figure 1: Study flow, illustrating the selection process for inclusion into the analysis

111x88mm (96 x 96 DPI)

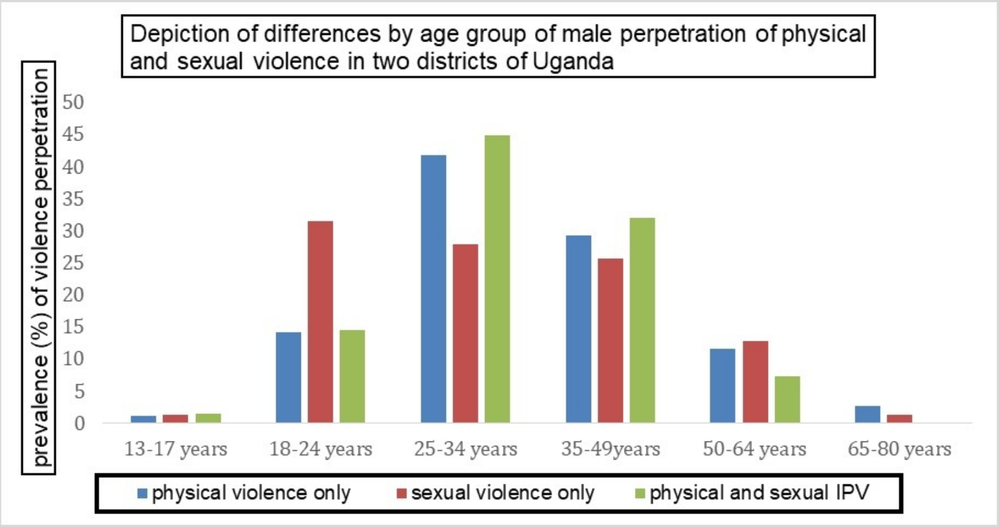

Figure 2: Depiction of lifetime prevalence by age group of male lifetime IPV perpetration in two districts of Uganda, May 2018 to July 2019.

154x81mm (150 x 150 DPI)

Online supplementary Appendix1:

Sensitivity analysis: Characteristics of the analysed sample (N=1314) and non-responders (N=321)

Variable	Analysed sample N (%)	Non-responders N (%)	p-value
**Study Strata**			
Wakiso communities	**578**	**179**	0.364
**Urban -Kazo**	206(35.6)	68(37.9)	
**Semi-urban -Lukwanga**	219(37.9)	62(34.6)	
**Rural -Sentema**	153(26.5)	49 (27.4)	
Hoima communities	**736**	**142**	0.210
**Urban –Ishaka_Kijungu**	243(33.0)	67(47.2)	
**Semi-urban-Butem**	198(25.5)	39(27.5)	
**Rural -Kitoba**	295(40.1)	42 (29.6)	
**Age (mean, SD), interquartile range(years)**	34 ±12.9, 24-42	15.8 ±3.4, 14-17	<0.05
**Education**			
**Primary school and below**	688(52.3)	149(46.4)	>0.001
**Secondary school**	477(36.3)	111(34.5)	
**Post-secondary**	149(11.3)	61(19.0)	
**Not married**	151(11.5)	0	
**Religion**			
**Christianity**	1068(81.3)	230(71.7)	0.02
**Non-Christians**	246(18.7)	91(28.3)	
**HIV status from blood sample**			
**Negative**	1224(94.3)	310(96.6)	<0.001
**Positive**	74(5.7)	11(3.4)	
**Past-year food insecurity**			
**No food insecurity**	995(75.7)	240(74.9)	0.891
**Low food insecurity**	272(20.7)	74(23.2)	
**High food insecurity**	47(3.6)	7(1.9)	

STROBE Statement—Checklist of items that should be included in reports of ***cross-sectional studies***

	Item No	Recommendation	Page No
**Title and abstract**	1	(*a*) Indicate the study's design with a commonly used term in the title or the abstract	✓ Page1-2
		(*b*) Provide in the abstract an informative and balanced summary of what was done and what was found	✓ Page 2
**Introduction**			
Background/rationale	2	Explain the scientific background and rationale for the investigation being reported	✓ Page 4-6
Objectives	3	State specific objectives, including any prespecified hypotheses	✓ Page6
**Methods**			
Study design	4	Present key elements of study design early in the paper	✓ Page6-7
Setting	5	Describe the setting, locations, and relevant dates, including periods of recruitment, exposure, follow-up, and data collection	✓ Page7
Participants	6	(*a*) Give the eligibility criteria, and the sources and methods of selection of participants	✓ Page7-8
Variables	7	Clearly define all outcomes, exposures, predictors, potential confounders, and effect modifiers. Give diagnostic criteria, if applicable	✓ Page8-10
Data sources/ measurement	8*	For each variable of interest, give sources of data and details of methods of assessment (measurement). Describe comparability of assessment methods if there is more than one group	✓ *Page9-10*
Bias	9	Describe any efforts to address potential sources of bias	✓ Page7-8
Study size	10	Explain how the study size was arrived at	✓ Page7
Quantitative variables	11	Explain how quantitative variables were handled in the analyses. If applicable, describe which groupings were chosen and why	✓ Page 9-11
Statistical methods	12	(*a*) Describe all statistical methods, including those used to control for confounding	✓ Page 11
		(*b*) Describe any methods used to examine subgroups and interactions	x
		(*c*) Explain how missing data were addressed	x
		(*d*) If applicable, describe analytical methods taking account of sampling strategy	N/A
		(*e*) Describe any sensitivity analyses	✓ Page8
**Results**			
Participants	13*	(a) Report numbers of individuals at each stage of study—eg numbers potentially eligible, examined for eligibility, confirmed eligible, included in the study, completing follow-up, and analysed	✓ Page8
		(b) Give reasons for non-participation at each stage	x
		(c) Consider use of a flow diagram	x

For peer review only - http://bmjopen.bmj.com/site/about/guidelines.xhtml
For peer review only

Descriptive data	14*	(a) Give characteristics of study participants (eg demographic, clinical, social) and information on exposures and potential confounders	✓ Page13
		(b) Indicate number of participants with missing data for each variable of interest	NA
Outcome data	15*	Report numbers of outcome events or summary measures	✓ Page14
Main results	16	(*a*) Give unadjusted estimates and, if applicable, confounder-adjusted estimates and their precision (eg, 95% confidence interval). Make clear which confounders were adjusted for and why they were included	✓ Page15-16
		(*b*) Report category boundaries when continuous variables were categorized	NA
		(*c*) If relevant, consider translating estimates of relative risk into absolute risk for a meaningful time period	NA
Other analyses	17	Report other analyses done—eg analyses of subgroups and interactions, and sensitivity analyses	x
**Discussion**			
Key results	18	Summarise key results with reference to study objectives	✓ Page18
Limitations	19	Discuss limitations of the study, taking into account sources of potential bias or imprecision. Discuss both direction and magnitude of any potential bias	✓ Page20-21
Interpretation	20	Give a cautious overall interpretation of results considering objectives, limitations, multiplicity of analyses, results from similar studies, and other relevant evidence	✓ Page18-20
Generalizability	21	Discuss the generalizability (external validity) of the study results	✓ Page20
**Other information**			
Funding	22	Give the source of funding and the role of the funders for the present study and, if applicable, for the original study on which the present article is based	✓ Page22

*Give information separately for exposed and unexposed groups.

Note: An Explanation and Elaboration article discusses each checklist item and gives methodological background and published examples of transparent reporting. The STROBE checklist is best used in conjunction with this article (freely available on the Web sites of PLoS Medicine at http://www.plosmedicine.org/, Annals of Internal Medicine at http://www.annals.org/, and Epidemiology at http://www.epidem.com/). Information on the STROBE Initiative is available at www.strobe-statement.org.

For peer review only

For peer review only - http://bmjopen.bmj.com/site/about/guidelines.xhtml

BMJ Open

Household food insecurity and its association with self-reported male perpetration of intimate partner violence: a survey of two districts in Central and Western Uganda

Journal:	*BMJ Open*
Manuscript ID	bmjopen-2020-045427.R2
Article Type:	Original research
Date Submitted by the Author:	11-Mar-2021
Complete List of Authors:	Awungafac, George; Karolinska Institute, Department of Global Public Health<br>Mugamba, Stephen; Africa Medical and Behavioural Science Organization<br>Nalugoda, Fred; Africa Medical and Behavioural Science Organization<br>Sjöland, Carl Fredrik; Karolinska Institute, Department of Global Public Health<br>Kigozi, Godfrey; Africa Medical and Behavioural Science Organization<br>Rautiainen, S; Karolinska Institute, Department of Global Public Health<br>Malyabe, Robert; Africa Medical and Behavioural Science Organization<br>Ziegel, Leo; Karolinska Institute, Department of Global Public Health<br>Nakigozi, Gertrude; Africa Medical and Behavioural Science Organization<br>Nalwoga, Grace; Africa Medical and Behavioural Science Organization<br>Kyasanku, Emmanuel; Africa Medical and Behavioural Science Organization<br>Nkale, James; Africa Medical and Behavioural Science Organization<br>Watya, Stephen; Africa Medical and Behavioural Science Organization<br>Ekström, Anna; Karolinska Institute, Department of Global Public Health<br>Kågesten, Anna; Karolinska Institute, Department of Global Public Health
**Primary Subject Heading**:	Global health
Secondary Subject Heading:	Epidemiology, Public health, Health policy, HIV/AIDS
Keywords:	Public health < INFECTIOUS DISEASES, Epidemiology < INFECTIOUS DISEASES, HIV & AIDS < INFECTIOUS DISEASES, MENTAL HEALTH

SCHOLARONE™ Manuscripts

I, the Submitting Author has the right to grant and does grant on behalf of all authors of the Work (as defined in the below author licence), an exclusive licence and/or a non-exclusive licence for contributions from authors who are: i) UK Crown employees; ii) where BMJ has agreed a CC-BY licence shall apply, and/or iii) in accordance with the terms applicable for US Federal Government officers or employees acting as part of their official duties; on a worldwide, perpetual, irrevocable, royalty-free basis to BMJ Publishing Group Ltd ("BMJ") its licensees and where the relevant Journal is co-owned by BMJ to the co-owners of the Journal, to publish the Work in this journal and any other BMJ products and to exploit all rights, as set out in our licence.

The Submitting Author accepts and understands that any supply made under these terms is made by BMJ to the Submitting Author unless you are acting as an employee on behalf of your employer or a postgraduate student of an affiliated institution which is paying any applicable article publishing charge ("APC") for Open Access articles. Where the Submitting Author wishes to make the Work available on an Open Access basis (and intends to pay the relevant APC), the terms of reuse of such Open Access shall be governed by a Creative Commons licence – details of these licences and which Creative Commons licence will apply to this Work are set out in our licence referred to above.

Other than as permitted in any relevant BMJ Author's Self Archiving Policies, I confirm this Work has not been accepted for publication elsewhere, is not being considered for publication elsewhere and does not duplicate material already published. I confirm all authors consent to publication of this Work and authorise the granting of this licence.

For peer review only

Household food insecurity and its association with self-reported male perpetration of intimate partner violence: a survey of two districts in Central and Western Uganda

George Awungafac[1*], Stephen Mugamba[2], Fred Nalugoda[2], Carl Fredrik Sjöland[1], Godfrey Kigozi[2], Rautiainen, S[1], Robert Bulamba Malyabe[2], Leo Ziegel[1], Gertrude Nakigozi[2], Grace Kigozi Nalwoga[2], Emmanuel Kyasanku[2], James Nkale[2], Stephen Watya[2], Anna Mia Ekström[1], Anna Kågesten[1]

Author affiliations

1. Department of Global Public Health, Karolinska Institutet, Stockholm Sweden

2. Africa Medical and Behavioural Sciences Organisation, Kampala, Uganda

*Corresponding author: **awungafacg@yahoo.com**

peer review only

For peer review only - http://bmjopen.bmj.com/site/about/guidelines.xhtml

Abstract

Objectives: This study aimed to determine the lifetime prevalence of male perpetrated intimate partner violence (IPV), and to assess the association with food insecurity, sociodemographic factors, and health-risk behaviours in Uganda in the year preceding COVID-19--associated lockdowns.

Design: Population-based, cross-sectional household survey.

Setting: Urban, semi-urban and rural communities of the Wakiso and Hoima Districts in Uganda.

Participants: A total of N=2014 males aged 13-80 years participated in the survey. The current study included males who reported having ever been in a sexual union and responded to the IPV questions (N=1314).

Measures: Data was collected face-to-face from May 2018 to July 2019 using an interviewer-mediated questionnaire. Lifetime IPV perpetration was measured as "no physical and/or sexual IPV", "physical" versus "sexual violence only", and "physical and sexual violence". Past-year food insecurity was measured through the Food Insecurity Experience Scale and categorized into "none", "low" and "high". Multinomial logistic regression was used to determine the crude and adjusted relative risk ratios (aRRR) of IPV perpetration in relation to self-reported food insecurity, adjusting for sociodemographic and health-risk behaviours.

Results: The prevalence of self-reported lifetime IPV perpetration was 14.6% for physical and 6.5% for sexual violence, while 5.3% reported to have perpetrated both physical and sexual IPV. Most (75.7%) males reported no food insecurity, followed by low (20.7%) and high (3.6%) food insecurity. In adjusted models, food insecurity was associated with increased risk of having perpetrated both physical and sexual violence (aRRR=2.57, 95%CI; 1.52-4.32). IPV perpetration was also independently associated with having had more than one lifetime sexual partner and drinking alcohol, but not with education level or religion.

For peer review only - http://bmjopen.bmj.com/site/about/guidelines.xhtml

Conclusion: This study suggests that food insecurity is associated with male IPV perpetration, and more efforts are needed to prevent and mitigate the expected worsening of this situation as a result of the COVID-19 pandemic.

Key words: Intimate Partner Violence, Men, Food Insecurity, Uganda.

Strengths and limitations of the study

- This is the first study to investigate the association between lifetime male perpetration of intimate partner violence (IPV) with food insecurity in Uganda.
- We included a population-based, diverse and representative sample of participants from different geographic areas (rural, semi-urban and urban) in Uganda.
- The cross-sectional nature of the study precludes any conclusion about the temporal association between IPV and food insecurity.
- Gender-based violence including IPV is a sensitive topic, and may be subject to under-reporting and social desirability bias. Nonetheless, this pre-pandemic assessement serves as an important baseline for furher studies of the link between food inscerutity and IPV during and after COVID-19-related lockdowns in Uganda.

For peer review only

Introduction

Intimate partner violence (IPV) is a major global health problem and human rights violation. IPV consists of intentional and abusive attitudes in the form of physical, sexual and/or emotional offense and controlling behaviours within an intimate partnership (1). While IPV can take different forms, male perpetrated IPV is the most common form of violence against women (VAW) (2). Globally, one in three ever-partnered women report experiencing some form of physical and/or sexual violence in their lifetime, with the World Health Organization (WHO) for the Africa region (WHO AFRO) recording the highest prevalence (37%), followed by the Americas (29.8%) (3). The WHO multi-country study on VAW estimated that the prevalence of male IPV perpetration was 21.1% for physical violence only, 11.7% for sexual violence only, and 11.8% for both physical and sexual violence (4). Reports from diverse social and cultural contexts have demonstrated that IPV perpetration begins early in the life course, with most perpetrators reporting that they first engage in it during adolescence (5).

IPV carries multiple, well-established consequences for women's health and wellbeing, with femicide being the most extreme form (6,7). IPV is associated with increased risk of sexually transmitted infections (STI) including HIV (8,9), severe depressive symptoms and substance use and abuse, such as binge drinking (10). Furthermore, children born to women experiencing IPV are at higher risk of premature death, poor health outcomes, and emotional and behavioural problems later in life (11). Experiencing IPV has been shown to affect women and girls' social and economic empowerment (12), including decreased productivity at work, loss of employment opportunities and other important social engagement (13).

Uganda has one of the highest burdens of IPV in the world and women's experiences of IPV have been the main focus of research on VAW in the country. The 2014 Ugandan Demographic

For peer review only - http://bmjopen.bmj.com/site/about/guidelines.xhtml

and Health Survey (DHS) found that 47% of married women had experienced physical IPV in their lifetime, with 29% reporting lifetime sexual IPV (14). Slapping, hitting and beating pregnant women was found to be common, and has been shown to worsen during the course of pregnancy in Northern Uganda (15,16). Furthermore, the country's HIV incidence has been found to correlate with the frequency and duration of exposure to IPV (17).

While multiple intersecting factors drive male perpetration of VAW, there is growing evidence linking IPV with food insecurity, most often related to poverty (18,19), which is defined as "a household-level economic and social condition of limited or uncertain access to adequate food" (20). For example, a cross-sectional study in Kampala slums found that extreme physical violence perpetration and experience of such violence was common among a convenience sample of young men (14-24 years), and was found to correlate with reported hunger and alcohol consumption, illicit drug use, poor mental health status and parental neglect due to alcohol (21). In a report from South Africa by Gibbs et al., economic indicators of food insecurity, such as unemployment and low earnings in the past month, were associated with IPV perpetration by men (22). A small-scale study from Abidjan, Cote d'Ivoire revealed that women with severe forms of food insecurity were at higher risk of experiencing IPV (23). While studies from other regions such as Nepal or the Unites States have established a significant relationship between food insecurity and both IPV perpetration and experiences among women, few studies from sub-Saharan Africa exist on this topic (24,25).

According to the United Nations (UN), food insecurity is increasing in Uganda, rising from 24.1% in 2006 to 41% in 2018 (26). The ongoing COVID-19 crisis has contributed to this considerable increase in Ugandan food insecurity and, in line with predictions of the World Bank and the World Food Programme, is likely to continue to do so (27,28). Likewise, there is great concern

For peer review only - http://bmjopen.bmj.com/site/about/guidelines.xhtml

that IPV is increasing across the world as a consequence of social isolation, povery and despair due to COVID-19-related lockdowns, social and financial hardships (29).

Male IPV perpetration against women is a complex, multi-level, social, economic and structural problem, one rooted in unequal gender norms and systems of power. In most cultural settings, a gendered order exists that favours stereotypically masculine men's dominance over women as well as over other more marginalised masculinities that do not live up to the norms of being a "real" man (30). IPV can thus be viewed both as a symptom of and a tool to achieve this type of "hegemonic" masculinity and power (31). Being unable to provide food for one's self or household could be interpreted as failing to meet stereotypical masculine norms, thereby forcing men to use alternative strategies (such as violence) to demonstrate their manhood (19). Mental health issues (depression or anxiety), which occur when concerns about food availability are accompanied by poor coping mechanisms such as alcohol consumption, can further lead to IPV as demonstrated by Hatcher and colleagues in peri-urban South Africa (19). Furthermore, the concept of "patriarchal risk" (32) can help to theoretically explain the cultural and societal dependence of women on male family members for food and protection, and trandtional gender roles are closely linked to all dimensions of food insecurity; access, availability, stability and utilzation. Although women tend to have less control over the household budget, they are often held responsible for feeding the family and blamed if they fail to provide food on the table, leading to different harmful coping strategies such as transactional sex, which in turn increases their risk of HIV (33).

Given the increasing reports of food insecurity in Uganda (26) and the lack of existing literature in low- and-middle income sub-Saharan African settings, it is important to investigate the potential link between food insecurity and male perpetrated IPV in the country. The primary aims for the present study were to determine the prevalence of lifetime male IPV perpetration among

For peer review only - http://bmjopen.bmj.com/site/about/guidelines.xhtml

a representative sample of males in two Ugandan districts, and to assess its association with food insecurity. The secondary aim was to determine whether the strength of the association between food insecurity and male IPV perpetration is affected by socio-demographic factors and health-risk behaviours in the urban, semi-ruban and rural populations under study in central Uganda. The evidence gained through this study can help inform programs and policies to prevent and better respond to IPV in Uganda and beyond, something that likely will be needed more than ever as we enter into the post-pandemic era.

Methods

Study design

Data was collected between May 2018 and July 2019 as part of a cross-sectional baseline survey of a longitudinal open population-level cohort established by the Africa Medical and Behavioural Sciences Organization (AMBSO) to conduct population health surveillance (PHS) in the Wakiso and Hoima districts of Uganda. In brief, the AMBSO PHS aims to generate evidence-based data in order to inform policy on the health status of the population through periodic monitoring of disease trends and determinants of health. The cohort includes males and females aged 13-80 years in the study communities and collects yearly data on sociodemographic factors, violence including IPV, non-communicable diseases (diabetes, hypertension, cancers, etc.), communicable diseases (such as HIV and STIs), food and nutrition, immunization of children, health risks (alcohol use, illicit drug consumption, number of sexual partners, and male circumcision) and mental health. The current study uses the baseline data with a focus on men's lifetime perpetration of IPV in the study communities.

For peer review only - http://bmjopen.bmj.com/site/about/guidelines.xhtml

Study communities

With a population of nearly 2 million, Wakiso district lies in the central region of Uganda and surrounds parts of the capital, Kampala. Hoima district, located in the Bunyoro Region of mid-western Uganda, has a smaller population of roughly 570,000 individuals. The selection of these two districts was purposeful, based on their common and diverse characteristics. Wakiso has a high migrant worker population and is known to be a hotspot for sex workers and men who have sex with men. The discovery of oil in Hoima and increasing mining activities attract migrant workers, sex workers and fisher folks – populations known to have high risk of HIV (34). Each district was stratified into urban, semi-urban and rural areas. Residents in all urban and semi-urban areas, and those in a randomly selected rural community (representing the rural sub-counties), were included in the baseline survey.

Study population and sampling

The study population comprised of males aged 13-80 years in the study communities. A lower age limit of 13 years was considered because of the high prevalence of early sexual debut in Uganda (35). This study sample was defined as males who reported ever having a sexual relationship. In each study community, the sampling frame included all households. In each household, all males aged 13-80 years were considered potential participants. Data from the Ugandan population census suggested that the total populations of male inhabitants in Wakiso and Hoima districts were 949,035 and 287,906 respectively (36). In determining the sample size, the study population males aged 13-80 years was estimated to be 65% of the total male population in each district, i.e 621,617 and 188,578 in Wakiso and Hoima, respectively. The Uganda DHS 40% estimate of male IPV perpetration (35) and the study population were entered into Epin Info (version 7.2.3.0) STATCalc tool to estimate the study sample. A minimum sample

For peer review only - http://bmjopen.bmj.com/site/about/guidelines.xhtml

of N=738 was needed to estimate the association between food insecutrity and IPV perpetration, with 80% power at the alpha=0.05 level. To be eligible, participants had to be able and willing to respond to the survey questions and to provide informed consent. The full survey sample consisted of N=2014 male participants who were recruited into the AMBSO PHS cohort. Males who reported not ever having been in a sexual union (N=379) and those who abstained from responding to questions on male IPV perpetration (N=321) were excluded (Figure 1).

Even though the individual response rate was 80.4%, the final analysis sample (N=1314) was near double the estimated sample of N=738 participants needed to estimate the association. This 80.4% response rate is similar to that of a multi-country survey on male IPV perpetration in Asia (4). A sensitivity analysis comparing the characteristics of those who responded to the IPV questions and those who did not respond is included in online supplementary appendix 1. The sensitivity analysis revealed that, in comparison with those who responded to the IPV questions, non-responders were more likely to be young, Christian and of lower educational level. There was no significant difference in food insecurity among non-responders and responders. Any observed differences did not affect the findings as the final sample was more than double the minimum estimated sample needed to establish an association.

Procedures

Data was collected in the baseline using a structured questionnaire covering ten domains: sociodemographics; first sex experience; family planning practice; marital status and practices; food supply and food security; IPV; HIV testing services; HIV care and treatment services; prostate cancer; and behaviour risk. Trained data collectors of the same sex as participants collected the data via face-to-face interviews, using tools translated into local languages (Luganda in Wakiso and Runyoro in Hoima).

For peer review only - http://bmjopen.bmj.com/site/about/guidelines.xhtml

In addition to these survey questions, the PHS collected 10 millilitres of venous blood samples that were used to determine the HIV and syphilis status of participants, and aliquots stored for other future studies. HIV status was determined following the Ugandan Ministry of Health rapid test algorithm (37), and the rapid plasma regain serological test was used to determine syphilis status of the participants.

Dependent variables

The dependent variable was adapted from the WHO multi-country study on VAW (3) , and defined as lifetime self-reported perpetration of physical and/or sexual violence against an intimate (sexual) partner. Following the approach used by Fulu et al (4), the outcome was grouped into four independent and unordered categories: 1) no perpetration of physical and/or sexual violence (no IPV); 2) physical IPV perpetration only; 3) sexual IPV perpetration only; and 4) both physical and sexual IPV perpetration (Table 1). The "no IPV perpetration" category was used as the reference group, referring to males reporting to never have perpetrated physical and/or sexual violence against their intimate sexual partner. The "physical violence only" category consisted of males reporting to have perpetrated one or more forms of physical violence, but who had never used any sexual violence; "sexual violence only" was coded using the same approach but referring to one or more forms of sexual violence. Males who reported to having perpetrated both physical and sexual violence in their lifetime were grouped into the "physical and sexual violence" category. Lifetime estimates were used in this study in order to capture all self-reports of IPV perpetration.

Table 1. Definition and measurement of lifetime IPV perpetration (dependent variable)

Type of violence perpetration	**Have you ever done any of the following to your sexual partner?**

For peer review only - http://bmjopen.bmj.com/site/about/guidelines.xhtml

**Physical violence only ("yes" to ≥1 indicator)**	-	Push, slap or hold partner down?
	-	Punch with fist or with something that could hurt partner?
	-	Kick or drag partner?
	-	Tried to strangle or burn partner?
	-	Attacked partner with a knife, gun, or other type of weapon?
**Sexual violence only ("yes" to ≥1 indicator)**	-	Force her to perform sexual acts against partner's will?
	-	Used threats to force her to have sex when she did not want to?
	-	Physically force her to have sex against her wish?
**Physical and sexual violence ("yes" to ≥1 indicators of both)**	-	Perpetration of both physical violence and sexual violence
**No IPV perpetration**	-	No perpetration of both physical violence and sexual violence

Independent variables

Food insecurity

The main independent variable was past-year food insecurity, assessed using an adapted version of the Food Insecurity Experience Scale (FIES) questions (38) in which six selected items were used to evaluate the household food situation during the preceding 12 months (Table 2).

Table 2: Assessment questions and coding for the measurement and classification of food insecurity among in the study.

Question	Responses	Coding of responses
Q.1 In the past 12 months, were there months in which you did not have enough food to meet your family's needs?	Yes/ No	Yes=1, No=0
Q.2 In the last 12 months, have you or other adults in your household withheld a meal because there was not enough food?	Yes/ No	Yes=1, No=0
Q.3 In the last 12 months, did you or other adults in your household ever not eat for a whole day because there wasn't enough food?	Yes/ No	Yes=1, No=0
Q. 4 In the last 12 months, did you ever *cut the size of any of the children's meals* because there wasn't enough food?	Yes/No	Yes=1, No=0
Q. 5 In the last 12 months, how often were you worried that food would run out?	Always/Sometimes/Never	Always=1, Sometimes=1, Never=0

For peer review only - http://bmjopen.bmj.com/site/about/guidelines.xhtml

Q. 6 If yes in Q.5, How often did this happen?	Almost every month/ sometimes but not every month/ only 1 or 2 months/Don't remember	Almost every month=1, sometimes but not every month=1, only 1 or 2 months=0, don't remember=0

Source: Food Insecurity Experience Scale (FIES)

In line with international definitions (39), negative responses to assessment questions such as "no" "never" "only 1 or 2 months" were coded as "0" (referring to those who reported having minor access problems for food during the preceding 12 months), while positive or affirmative responses such as "yes", "always", "almost every month", "some months but not every month" were coded as "1" (referring to having problems with access to adequate food during the preceding 12 months). All codes were summed to obtain a total score of up to six (i.e. 0-6) for food insecurity, with higher scores indicating the extent of food insecurity. As adapted from international approaches (39), participants were categorised into the following categories: no food insecurity (0-1), low food insecurity (2-4) and high food insecurity (hunger) (5-6). Due to the small proportion reporting high food insecurity (3.6%), low food insecurity and high food insecurity were merged during the modelling procedures in line with previous studies (19), i.e. comparing food insecurity against food security in line with previous studies.

Sociodemographic and health-risk behaviours

Other important independent variables included sociodemographic indicators such as: study district (urban/semi-urban/rural); age; education level (categorized into primary school and below, secondary school and post-secondary); marital status (married or in a union/not married); religion (categorized into Christian, non-Christians); and having living children (yes/no). Health-risk behaviours included: number of lifetime sexual partners (one partner/two or more partners); past-year condoms use (yes/no); use of illicit drugs (yes/no); alcohol consumption (yes/ no); and male circumcision (yes/no). The choice of health-risk behaviours was based on previous studies

For peer review only - http://bmjopen.bmj.com/site/about/guidelines.xhtml

indicating number of sexual partners as well as (low) condom use (40–42) and male circumcision (42) as risk factors for IPV.

Statistical analysis

We first conducted descriptive analyses to explore the distribution of variables and to identify outliers. The analysis in this report was not weighted. Missing values for covariates were replaced using multiple imputation assuming random missingness. Pearson's Chi-squared (Chi2) test and Fishers exact test were used to compare proportions with α set at 5%. Next, we performed bivariate multinomial logistic regression (MLR) to determine the crude relative risk ratios (RRR) and 95% confidence intervals (CI) of IPV perpetration in relation to the independent variables. We followed the approach used by Fulu et al (4) and used "No IPV perpetration" as the base outcome against which all other outcomes were compared, assuming that the association of food insecurity is different for each typology of violence. This type of regression produces RRR, which are the exponentiated coefficients of the regression, and explain the relative effect of the independent variable on the outcomes using one of the outcomes as a base outcome. A full model was built to explain the associated factors to IPV perpetration in relation to both food insecurity, health-risk behaviours and sociodemographic background. The full model (adjusting for food insecurity and sociodemographic and health-risk behaviours) included all the variables from the bivariate analysis, irrespective of significance levels, that are theoretically known to be associated to IPV perpetration. Variables for the final adjusted model were selected through backward elimination using a maximum likelihood ratio (LR) test. All analyses were conducted using STATA version 16 (StataCorp), while Microsoft Office Excel was used to design figures.

For peer review only - http://bmjopen.bmj.com/site/about/guidelines.xhtml

Ethical considerations

The AMBSO PHS cohort study received ethical approval from the Clarke International University Ethics Committee (Ref:CIUREC/0059) as the local Institutional Review Board (IRB) of record, and clearance from the Ugandan Council of Science and Technology (Ref:SS4468). Clearance was obtained from both districts to conduct the study. All participants provided a written informed consent /assent before participating in the study. With the exception of emancipated minors (under 18 years) who (like adults) gave their own written or oral assent in addition to written, parental/guardians consent was obtained. Data was collected by a study team trained and certified in Human subject protection and Good clinical Practice. Interviews were conducted in private. Unique identification codes were used in all computerized data, and the lists that linked participants' names to the codes were accessible only to senior project staffs in password protected computers. Participants who tested positive for HIV infection and syphilis were linked to specialized care while those who needed other health support services were referred to health facilities of their choice.

Patients and Public Involvement statement

The study team worked hand in hand with community health workers, local community leaders and a community-research advisory structure to develop and clarify a common understanding of the aims of the population-based survey, the design and operational aspects as well as how results will be disseminated. As part of this "patient and public involvement" effort, a consensus was reached with the advisory committee on a plan to increase general public awareness of the study and health issues that were deemed areas of PHS focus.

For peer review only - http://bmjopen.bmj.com/site/about/guidelines.xhtml

Results

Sample characteristics

Table 3 shows the characteristics of the study participants. The mean age of respondents was 34 years (SD; ±12.9). Food insecurity was found to affect one in four men, with 20.7% and 3.6% categorized as low versus high food insecurity, respectively. About one third and 50% of men reported having had multiple sex partners and used alcohol in the past year, respectively; and approximately 6% tested positive for HIV.

Table 3. Sample characteristics of male respondents in the two study districts in Uganda (N=1314)

Variable	n	%
Age (mean, SD)	34 ±12.9	
Education		
Primary and below	688	52.4
Secondary school	477	36.3
Post-secondary	149	11.3
Marital status		
Married or in a union	1163	88.5
Not married	151	11.5
Have any living children		
Yes	999	76.2
No	312	23.8
Religion		
Christianity	1068	81.3
Non-Christian	246	18.7
Past-year food security		
No food insecurity	995	75.7
Low food insecurity	272	20.7
High food insecurity	47	3.6
Score (mean, SD)	1.48 ±1.05	
Number of lifetime sexual partners		
One partner	919	69.9
Two or more partners	395	30.1
Current use of condoms		
Yes	519	39.5
No	795	60.5
HIV status from blood sample		
Negative	1224	94.3
Positive	74	5.7
Past-year alcohol use		

For peer review only

For peer review only - http://bmjopen.bmj.com/site/about/guidelines.xhtml

Yes	677	48.5
No	640	51.5
Circumcised		
Yes	659	50.2
No	655	49.8
Past-year use of illicit drugs		
Yes	43	3.3
No	1271	96.7

Prevalence of lifetime IPV perpetration

Table 4 presents the prevalence of perpetration of different forms of IPV against an intimate (sexual) partner by male participants. Most (73.6%) males reported never having perpetrated any form of IPV. The prevalence of self-reported physical IPV perpetration ranged from 19.3% (pushing, slapping, holding down) to 0.9% (attacked partner with a knife, gun or other weapon), with 14.6% having perpetrated any form of physical violence only. Lifetime perpetration of sexual IPV ranged from 8.2% (threatened or pressured partner into sex when unwanted) to 5.4% (physically forced partner to have sex) and 2.4% (used other types of force to have sex), with 6.5% of males reporting having perpetrated any form of sexual IPV only. In total, 5.3% of males reported previous perpetration of both physical and sexual IPV.

Table 4: Prevalence of self-reported lifetime perpetration of IPV against a sexual partner among ever-partnered males aged 13-80 years in two districts of Uganda, May 2018 to July 2019

	**Lifetime IPV perpetration (N=1314)**	
	n	%
**Physical violence (any form)**	261	19.9
Push, slapped or held down partner	253	19.3
Kicked or dragged partner	54	4.1
Tried to strangle partner	19	1.5
Attacked partner with a knife, gun or other weapon	12	0.9
Punch with fist or with something that could hurt partner?	61	4.6
**Sexual violence (any form)**	155	11.8
Threatened or pressured partner into sex when unwanted	108	8.2
Physically forced partner into sex	70	5.4
Used other ways to force partner to perform sexual acts when unwanted	31	2.4
**Any physical or sexual violence**	347	26.4

For peer review only - http://bmjopen.bmj.com/site/about/guidelines.xhtml

**Physical IPV perpetration only**	192	14.6
**Sexual IPV perpetration only**	86	6.5
**Both physical and sexual IPV perpetration**	69	5.3
**No IPV perpetration**	967	73.6

Figure 2 shows the prevalence of self-reported IPV perpetration across different age groups . As can be seen, the prevalence of physical violence only and both physical and sexual violence was highest among 25-34-year-olds compared to the other age-groups ($p<0.001$), with perpetration of sexual violence only being most commonly reported by young males (18-24 years) and less common after age 34 ($p=0.517$).

Factors associated with IPV perpetration

Bivariate analysis

In bivariate analysis, food insecure participants had an RRR of 2.71 (95% CI 1.64-4.46) to have perpetrated both physical and sexual violence, but no significantly higher risk to have perpetrated physical vs. sexual violence only, respectively (Table 5). Compared to the youngest age group 13-24 years, young adult participants' (25-34 years) had a RRR of 2.59 (95%CI 1.64-4.07) for perpetrating physical violence only, and 2.64 (95%CI 1.30-5.36) for both physical and sexual violence, and the RRR for those aged 35+ years was 2.16 (95%CI 1.38-3.38) for physical violence. Being married was associated with a lower relative risk of having perpetrated sexual violence only. Having living children and reporting no condom use was associated with perpetration of physical violence only. Reported use of illicit drugs during the preceding year was associated with perpetration of physical violence only and both physical and sexual violence. There was no association between education level with IPV perpetration.

For peer review only - http://bmjopen.bmj.com/site/about/guidelines.xhtml

Table 5: Crude Relative Risk Ratios (RRR) of factors associated with male IPV perpetration in two districts of Uganda (baseline outcome=no IPV) (N=1314) 2019

Variable	N	No IPV	Physical violence only			Sexual violence only			Both Physical and sexual violence		
		N (%)*	N (%)*	RRR, 95% CI	p-value	N (%)*	RRR, 95% CI,	p-value	N (%)*	RRR, 95% CI,	p-value
Age group(years)											
13-24 (Ref)	353	285(80.7)	29(8.2)			28(7.9)			11(3.1)		
25-34	439	304(69.3)	80(18.2)	2.59(1.64-4.07)	0.001	24(5.5)	0.80(0.46-1.42)	0.451	31(7.1)	2.64(1.30-5.36)	0.007
35+	522	378(72.4)	83(15.9))	2.16(1.38-3.38)	0.001	34(6.5)	0.92(0.54-1.54)	0.741	27(5.2)	1.85(0.90-3.79)	0.093
Education											
Primary or below(Ref)	688	499(72.5)	106(15.4)			49(6.7)			37(5.4)		
Secondary school	477	349(73.2	68(14.3)	0.92(0.66-1.28)	0.612	34(7.1)	1.05(0.66-1.68)	0.815	26(5.5)	1.0(0.60-1.69)	0.986
Post-secondary	149	119(79.9)	18(12.1)	0.71(0.42-1.21)	0.216	6(4.0)	0.55(0.23-1.31)	0.176	6(4.0)	0.68(0.28-1.65)	0.393
Religion											
Islam and others (Ref)	242	186(76.9)	24(9.9)			16(6.6)			16(6.6)		
Christian religion	1072	781(72.8)	168(15.7)	1.66(1.06-2.63)	0.028	70(6.5)	1.04(0.59-1.83)	0.887	53(4.9)	0.79(0.44-1.41)	0.424
Marital status											
Not married (Ref)	151	109(72.2)	15(9.9)			18(11.9)			9(5.9)		
Married	1163	858(73.8)	177(15.2)	1.50(0.85-2.63)	0.159	68(5.9)	0.48(0.27-0.84)	0.010	60(5.2)	0.85(0.41-1.75)	0.655
Have living children											
No (Ref)	312	258(82.7)	18(5.7)			25(8.0)			11(3.5)		
Yes	999	708(70.9)	172(17.2)	3.48(2.10-5.77)	<0.001	61(6.1)	0.89(0.54-1.45)	0.636	58(5.8)	1.92(0.99-3.71)	0.053
Past-year Alcohol consumption											
No (Ref)	637	511(80.2)	74(11.6)			32(5.0)			20(3.1)		
Yes	677	456(67.4)	118(17.4)	1.79(1.30-2.45)	<0.001	54(7.9)	1.89(1.20-1.98)	0.006	49(7.3)	2.74(1.61-4.68)	<0.001
Past year illicit drug											
No (Ref)	1271	948(74.6)	180(14.2)			83(6.5)			60(4.7)		
Yes	43	19(44.2)	12(27.9)	3.32(1.59-6.97)	0.001	3(6.9)	1.80(0.52-1.22)	0.351	9(20.9)	7.48(3.24-17.2)	<0.001
Food insecure											

For peer review only - http://bmjopen.bmj.com/site/about/guidelines.xhtml

Variable	N	No IPV	Physical violence only			Sexual violence only			Both Physical and sexual violence		
No food insecurity (Ref)	995	756(76.0)	139(14.1)			56(5.7)			31(3.2)		
Food insecure	319	214(67.1)	52(16.3)	1.31(0.92-1.86)	0.395	23(7.2)	1.28(0.77-1.22)	0.097	30(9.4)	2.71(1.64-4.46)	<0.001
Condom use in past 12 months											
No(Ref)	795	571(71.8)	130(16.4)			50(6.3)			44(5.5)		
Yes	519	396(76.3)	62(11.9)	0.69(0.49-0.96)	0.026	36(6.9)	1.04(0.66-1.62)	0.870	25(4.8)	0.82(0.49-1.36)	0.441
Circumcised											
No(Ref)	655	464(70.8)	107(16.3)			43(6.6)			41(6.3)		
Yes	659	503(76.3)	85(12.9)	0.73(0.54-1.00)	0.050	43(6.5)	0.92(0.59-1.43)	0.720	28(4.3)	0.63(0.38-1.03)	0.068
Number of lifetime sexual partner											
One (Ref)	919	791(76.3)	124(13.5)			55(6.0)			39(4.2)		
Two or more	395	266(67.3)	68(17.2)	1.44(1.04-2.01)	0.028	31(7.8)	1.48(0.94-2.36)	0.098	30(7.5)	2.03 (1.23-3.33)	0.005
Hoima district (Ref: Wakiso)	736	526(71.5)	112(15.2)	1.17(0.86-1.61)	0.912	55(7.5)	1.49(0.94-2.35)	0.679	43(5.8)	1.38 (0.84-2.29)	0.875

*Row percentage

For peer review only

For peer review only - http://bmjopen.bmj.com/site/about/guidelines.xhtml

Multivariable analysis

Table 6 illustrates the adjusted relative risk (aRRR) for IPV perpetration in relation to food insecurity, controlling for sociodemographic factors and health risk behaviours. Food insecurity remained significantly associated with self-reported perpetration of both physical and sexual violence (aRRR=2.57, 95%CI; 1.52-4.32). In terms of independent health risk-factors, having two or more lifetime sexual partners also remained associated with perpetration of physical violence only, and both physical and sexual violence. Any alcohol consumption remained associated with all typologies of IPV perpetration under study, and illicit drug use remained associated with physical violence and both physical and sexual violence. In terms of sociodemographics, having living children remained associated with perpetration of physical only, while being married appeared to be protective against perpetration of sexual violence only. Age and religion were not associated with any IPV perpetration in the adjusted model.

For peer review only

Table 6: Adjusted relative risk ratios (aRRR) of factors associated with male IPV perpetration in two districts of Uganda, (N=1314).

Variable[a]	Physical violence only*				Sexual violence only*				Both physical and sexual violence*			
	Crude RRR, 95% CI	p-value	aRRR, 95% CI	p-value	Crude RRR, 95% CI	p-value	aRRR, 95% CI	p-value	Crude RRR, 95% CI	p-value	aRRR, 95% CI	p-value
Food-insecurity (ref: no food insecurity)	1.31 (0.92-1.86	0.395	1.19 (0.82-1.73)	0.386	1.28 (0.77-2.12	0.097	1.33 (0.54-1.55)	0.235	2.71 (1.64-4.46)	<0.001	2.57 (1.52-4.32)	0.002
**Health-risk behaviours**												
Two or more sexual partners (ref: one partner)	1.0 (1.04-2.01)	0.028	1.64 (1.15-2.33)	0.012	1.48 (0.94-2.36)	0.098	1.38 (0.85-2.25)	0.243	2.03 (1.23-3.33)	0.005	1.97 (1.15-3.37)	0.016
Drink alcohol (ref: no)	1.79 (1.30-2.45)	<0.001	1.51 (1.07-2.12)	0.006	1.89 (1.20-2.98)	<0.001	1.89 (1.16-3.07)	0.002	2.74 (1.61-4.68)	<0.001	2.55 (1.42-4.58)	<0.001
Illicit drug use the past year (ref: no)	3.32 (1.59-6.97)	0.001	3.84 (1.73-8.52)	0.003	1.80 (0.52-6.22)	0.351	1.29 (0.36-4.58)	0.657	7.48 (3.24-17.2)	<0.001	4.80 (1.93-11.9)	0.005
**Sociodemographic factors**												
25-34 years (ref: 18-24 years)	2.59 (1.64-4.07)	0.001	1.37 (0.99-1.91)	0.069	0.80 (0.46-1.42)	0.451	0.84 (0.51-1.39)	0.132	2.64 (1.30-5.36)	0.007	1.66 (0.99-2.78)	0.079
35+ years	2.16 (1.38-3.38)	0.001	0.95 (0.53-1.68)	0.346	0.92 (0.59-1.83)-	0.741	0.80 (0.41-1.54)	0.325	0.79 (0.44-1.41)	0.093	0.40 (0.19-0.84)	0.025
Christian religion (ref: non-Christians)	1.66 (1.06-2.63)	0.028	1.53 (0.94-2.51)	0.127	1.04 (0.59-1.83)	0.887	0.82 (0.45-1.51)	0.203	0.79 (0.44-1.41)	0.424	0.61 (0.32-1.17)	0.156
Married (Ref: not married	1.50 (0.85-2.63)	0.159	1.34 (0.76-2.44)	0.321	0.48 (0.27-0.84)	0.010	0.49 (0.27-0.86)	0.021	0.85 (0.41-1.75)	0.655	0.81 (0.37-1.75)	0.217
Have children (ref: no)	3.48 (2.10-5.77)	<0.001	3.24 (1.91-5.52)	**0.011**	0.89 (0.54-1.45)	0.636	0.92 (0.55-1.56)	0.325	1.92 (0.99-3.71)	0.053	1.77 (0.87-3.61)	0.876

[a]Food insecurity was adjusted for age group, marital status, number of sexual partners, illicit drug use, past-year alcohol consumption, having living children and religion. *Reference/base outcome: no IPV perpetration.

For peer review only

For peer review only - http://bmjopen.bmj.com/site/about/guidelines.xhtml

Discussion

The current study set out to explore the prevalence of lifetime male IPV perpetration and its association with food insecurity as well as sociodemographics and health risk-behaviours in two Ugandan districts. We found that one in four men reported ever perpetrating any form of physical and/or sexual violence against their intimate partners, and 5.3% reported perpetrating both physical and sexual IPV. Past-year food insecurity was associated with male perpetration of both physical and sexual violence after adjusting for age group, religion, number of lifetime sexual partners, marital status, having living children alcohol use and illict drug use.

The prevalence of lifetime perpetration of IPV reported by this study are lower than that of other studies conducted in the region (19,43,44). Obtaining accurate data on the true burden of VAW is a global concern due to under reporting (45). It is likely that community awareness about laws and litigations in place to prevent VAW may undermine truthful responses to IPV survey questions. Nonethelss, a fair proportion of men in our survey did admit to IPV petpetration. In these new demographic surveillance sites and first round of the assessments, it is possible that the communities have not yet built sufficient trust with the survey teams to report very sensitive information and potential illegal behaviours. Comparing the prevalence estimates of this study with data from previous studies is therefore difficult, mainly due to the existence of limited research in a sub-Saharan African context, and the non-standardized ways in which surveys have assessed male IPV perpetration. For instance, although the 2006 Uganda DHS (14) found that 40% of males perpetrated IPV, this finding was solely based on reports of physical violence (e.g. partner hitting, slapping and kicking) without taking into account sexual IPV perpetration. Another large-scale study that focused on past-year perpetration (rather than lifetime) in the Rakai district of Uganda indicated a prevalence of 10.4% for physical violence and 3.1% for

For peer review only - http://bmjopen.bmj.com/site/about/guidelines.xhtml

sexual violence (42). In the present study, 19.3% of men reported ever slapping or pushing their intimate partners, an estimate that is similar to other countries including Ghana (17.4%) (43) and Vietnam (23.5%) (46), but lower than those obtained by the International Men and Gender Equality study in the Democratic Republic of Congo (45.1%) and Rwanda (39.1%) (44).

In this pre-pandemic assessment, one in four men reported to be food insecure and food insecurity was associated with almost three times the relative risk of having perpetrated both physical and sexual IPV. Our findings are consistent with results of a recent study from a peri-urban setting of South Africa, in which food insecurity doubled the odds of males' perpetration of IPV (19). The results from our study align with findings from Nepal (25) and USA (24) that food insecurity was more common among married women who had experienced physical violence from their intimate sexual partners; however, the causal relationships are uncertain. The World Food Program expects the number of people who are food insecure to increase dramatically as a consequence of COVID-19-related mobility restrictions and poverty, potentially driving another 135 million people worldwide onto the brink of starvation. Similarly, United Nations Populaton Fund warns of increases in IPV as a consequnve of social isolation, hopelssness and financial hardship (29). Thus, the pre-pandemic estimates presented in this study are expected to worsen and should serve as an important warning indicator for urgent mitigation efforts.

Previous research indicates that the association between food insecurity and IPV perpetration may be driven by mental health problems such as anxiety and depression that arise from concerns about food availability related to poverty and unemployment (19), which are also expected to increase as a results of the current pandemic. The influence of conservative gender norms is important, whereby the inability of men to provide for their partners and households affects their perceived masculinity and contributes to their use of violence (47). A qualitative study in Bangladesh found that inadequate food portions offered to men may trigger retaliatory

For peer review only - http://bmjopen.bmj.com/site/about/guidelines.xhtml

acts of violence against their wives, and that men could withhold resources used to acquire food as a form of power (48). Corroborated by previous research, the present study also found that men who had consumed alcohol in the past year and who have multiple lifetime sexual partners were at significantly higher risk of perpetrating IPV (4,49,50). The use of illicit drugs, though assumed to be under-reported, was also found to be associated with IPV perpetration after adjusting for confounding, in line with previous research (51). The association between food insecurity and IPV perpetration may thus be influenced and mediated by multiple factors previously examined, e.g. through structural equation models including poor mental health, gender attitudes, multiple partnerships, controlling behaviours and alcohol consumption (19,22).

Furthermore, we found that the prevalence of IPV perpetration varied by age group, with reports of sexual and physical IPV being more common among young adult males compared to older men, while in the adjusted models, being 35 years and older appears to confer a protective effect against perpetraton of IPV. These findings are in line with previous studies from the US (50,52). For young men, it is possible that a relative lack of relationship experiences, masculine identity seeking and vulnerability to peer-influence (50,52) increase their tendency to perpetrate violence (53). These findings have implications for the design of future preventive interventions for different sub-populations, especially men in the younger age groups.

Our findings highlight the need for early prevention of IPV that targets young men, and the necessity to investigate and respond to food insecurity within the framework of IPV prevention. Even though a temporal association between IPV and food insecurity is yet to be established, the findings of this study should serve as an alert to more initiatives to address IPV, particularly in light of an expected dramatic increase in both IPV and food insecurity reports as a consequences of the COVID-19 pandemic (29). Existing IPV preventive interventions in Uganda such as the SASA community based program (54) and the Program P initiative (55) may provide

For peer review only - http://bmjopen.bmj.com/site/about/guidelines.xhtml

concrete guidelines for efforts to engage more men in IPV preventive programmes. The SASA and Program P interventions also use comprehensive strategies (including HIV risk reduction) and should be strengthenedwith considerations for advocacy to include for food security measures as part of the packages to respond to IPV. There is an urgent need to adapt context-relevant interventions to address food insecurity, especially during the current pandemic with severe increases in both IPV and food insecurity warning and reports world-wide. This is of particular importance in Sub-Saharan Africa, where a huge surge in poverty rates and food insecurity is expected (56,57). Thus, our study suggests that IPV may become an even larger public health issue in Uganda and beyond in the near future. Potential interventions to reduce poverty and food insecurity could include: efforts to boost agricultural food production such as short-term provision of seeds and tools to rehabilitate farming and facilitating the acquisition of credits for small businesses; conditional cash transfers to women household heads to enable purchase of necessities and pay for health expenses; implementation of school fee waivers and school feeding programs; and the provision of food vouchers to enable purchase of food in local markets and the emergency distribution of food (58). Structural interventions to address IPV should be advocated for, including more discussion on gender norms and harmful masculinities in schools and communities, putting in place systems to prevent male violence perpetration, encouraging women to report incidences of IPV accompanied with a strengthening of legislation, training and monitoring of the police force and justice departments in IPV-related issues and set up support and counselling centres for women who experience IPV (59).

Limitations

This study is one of the first to investigate lifetime perpetration of different forms of physical and sexual violence among both younger and older men, and to examine their association with food insecurity and male IPV perpetration in a population-based, representative cohort in rural, urban

For peer review only - http://bmjopen.bmj.com/site/about/guidelines.xhtml

and semi-urban Uganda. Given the self-reported nature of the data on a sensitive topic like IPV, under-reporting of physical and sexual IPV as well as recall bias is possible. Some participants may have considered the questions to be too private and/or shameful to talk about, and some may have feared legal implications (given that IPV is illegal), or found it challenging to respond honestly. In light of this potential social desirability bias, the true rate of IPV perpetration is likely higher than reported in this first round of the assessments. In the future, exposure to and increased population trust in the data collectors in this reoccurring population-based survey may further improve the confidence in self-reporting IPV perpetration. Even though the minimum sample needed for this study was attained, a fairly high proportion of men did not respond to the IPV questions in the survey. The sensitivity analysis revealed, however, that this had no significant effect on the findings. Data on poverty indicators such as income levels and employment status were unfortunately lacking at individual level, but it can be assumed that food insecurity is a reflection of income level and the availability and access to livelihood resources. Finally, due to the cross-sectional nature of the data coupled with the measurement of food insecurity as past-year and IPV as lifetime, we were unable to establish a temporal association between food insecurity and IPV perpetration. Even though multivariate analysis was performed in this study, the likelihood of residual confounding still remains.

Conclusions

Although likely an underestimation, the reported prevalence of male IPV perpetration in these Ugandan communities was still sizeable – in particular among young men, and must be addressed by enhanced IPV preventive strategies and programming in schools and communities. Food insecurity was associated with both physical and sexual IPV, suggesting the need for integrated approaches to address VAW, poverty and food insecurity. Considering the impact of COVID-19 lockdowns and multiple reports on increased IPV, as well as expected huge

For peer review only - http://bmjopen.bmj.com/site/about/guidelines.xhtml

increases in poverty rates and food insecurity throughout Uganda, the need for preventive interventions is even greater than before. Longitudinal surveys are needed to investigate the temporal relationships and drivers of food insecurity in Uganda and beyond, and to determine its impact on different forms of violence.

Contributorship statement

The original ideas leading to the Population Health Surveillance study from which this manuscript is derived, were put together by SM, FN, GK, GN, SW and AME. GA and AK conceptualised the research questions for the current study. GA analysed the data, wrote the first draft of the study and oversaw the manuscript development process. SM, FN, SW and GK coordinated the participant recruitment as well as the data collection, management and entry. RBM, LZ, GN, GKN, EK and JN participated in actual data collection from the study participants. CFS and SR contributed in describing the measurement and classification of food insecurity. AK provided oversight to the study, providing edits, structure and scope to the study together with AME. All authors reviewed the drafts leading to this final manuscript.

Funding

This study was made possible by private funds from AMBSO directors, the Karolinska Institutet through a Swedish Research Council grant (grant number:2019-04474), and the Swedish Institute which supported the first author with an educational study grant (Ref:02205/2019).

For peer review only - http://bmjopen.bmj.com/site/about/guidelines.xhtml

Acknowledgement

We thank the staff of Africa Medical and Behavioral Sciences Organization (AMBSO); the PHS study participants; the local community leadership; and the Wakiso and Hoima Districts Directorates of Health services for supporting this work.

Conflict of interest

None declared

Data availability statement

The data for this study is strictly not available to the public. A reasonable request for the data can be made by contacting Stephen Mugamba (smugamba@gmail.com)

References

1. Krug EG et al., eds. World report on violence and health. Geneva, World Health Organization, 2002.

2. Richardson JR, Feder G, Coid J. Domestic violence affects women more than men. BMJ Br Med J. 2002;325(7367):779.

3. World Health Organisation. Global and regional estimates of violence against women: prevalence and health effects of intimate partner violence and non-partner sexual violence. Geneva; 2013.

4. Fulu E, Jewkes R, Roselli T, Garcia-Moreno C. Prevalence of and factors associated with male perpetration of intimate partner violence: findings from the UN Multi-country Cross-sectional Study on Men and Violence in Asia and the Pacific. Lancet Glob Heal. 2013 Oct 1;1(4):e187–207.

5. Peitzmeier SM, H MSP, Kågesten A, H MP, Acharya R, Ph D, et al. Intimate Partner Violence Perpetration Among Adolescent Males in Disadvantaged Neighborhoods Globally. 2016;1–7.

For peer review only - http://bmjopen.bmj.com/site/about/guidelines.xhtml

6. Martin PM, Carvajal N. Feminicide as 'act' and 'process': a geography of gendered violence in Oaxaca. Gender, Place Cult. 2016;23(7):989–1002.

7. Prieto-Carrón M, Thomson M, Macdonald M. No more killings! Women respond to femicides in Central America. Gend Dev. 2007;15(1):25–40.

8. Campbell JC, Baty ML, Ghandour RM, Stockman JK, Francisco L, Wagman J. The intersection of intimate partner violence against women and HIV/AIDS: a review. Int J Inj Contr Saf Promot. 2008;15(4):221–31.

9. Coker AL. Does physical intimate partner violence affect sexual health?: A systematic review. Vol. 8, Trauma, Violence, and Abuse. Sage PublicationsSage CA: Los Angeles, CA; 2007. p. 149–77.

10. Tsai AC, Wolfe WR, Kumbakumba E, Kawuma A, Hunt PW, Martin JN, et al. Prospective Study of the Mental Health Consequences of Sexual Violence Among Women Living With HIV in Rural Uganda. J Interpers Violence. 2015;31(8):1531–53.

11. Hossain MA, Sumi NS, Haque ME, Bari W. Consequences of Intimate Partner Violence Against Women on Under-Five Child Mortality in Bangladesh. J Interpers Violence. 2014;29(8):1402–17.

12. The World Bank. The Cost of Violence. Washington DC; 2009.

13. López-Sánchez MJ, Belso-Martínez JA, Hervás-Oliver JL. A Review of Economic Consequences and Costs of Male Violence Against Women. Indian J Gend Stud. 2019;26(3):424–34.

14. Ogland EG, Xu X, Bartkowski JP, Ogland CP. Intimate Partner Violence Against Married Women in Uganda. J Fam Violence. 2014 Nov 18;29(8):869–79.

15. Clarke S, Richmond R, Black E, Fry H, Obol JH, Worth H, et al. Intimate partner violence in pregnancy : sectional study from post- - conflict a cross- - northern Uganda. 2019;1–9.

16. Kaye DK, Mirembe FM, Bantebya G, Johansson A, Ekstrom AM. Domestic violence during pregnancy and risk of low birthweight and maternal complications: a prospective cohort study at Mulago Hospital, Uganda.

17. Kouyoumdjian FG, Calzavara LM, Bondy SJ, O'Campo P, Serwadda D, Nalugoda F, et al. Intimate partner violence is associated with incident HIV infection in women in Uganda. Aids. 2013;27(8):1331–8.

18. Food and Agricultural Organisation of the United Nation. How can food security interventions

For peer review only - http://bmjopen.bmj.com/site/about/guidelines.xhtml

contribute to reducing gender-based violence ?Issue Brief. 2017;

19. Hatcher AM, Stöckl H, McBride R-S, Khumalo M, Christofides N. Pathways From Food Insecurity to Intimate Partner Violence Perpetration Among Peri-Urban Men in South Africa. Am J Prev Med. 2019 May 1;56(5):765–72.

20. Food Insecurity: A Public Health Issue. Public Health Rep. 2016;131(5):655.

21. Swahn MH, Gressard L, Palmier JB, Kasirye R, Lynch C, Yao H. Serious violence victimization and perpetration among youth living in the slums of Kampala, Uganda. West J Emerg Med. 2012;13(3):253–9.

22. Id AG, Jewkes R, Willan S, Washington L. Associations between poverty , mental health and substance use , gender power , and intimate partner violence amongst young (18- 30) women and men in urban informal settlements in South Africa : A cross-sectional study and structural equation model. 2018;(January 2017):1–19.

23. Fong S, Gupta J, Kpebo D, Falb K. Food insecurity associated with intimate partner violence among women in Abidjan , Cote d ' Ivoire An examination of femicides in Peru between 2009 and 2014. 2016;341–2.

24. Ricks JL, Cochran SD, Arah OA, Williams JK, Seeman TE. Food insecurity and intimate partner violence against women: Results from the California Women's Health Survey. Public Health Nutr. 2016;19(5):914–23.

25. Diamond-Smith N, Conroy AA, Tsai AC, Nekkanti M, Weiser SD. Food insecurity and intimate partner violence among married women in Nepal. J Glob Health. 2019 Jun;9(1):010412.

26. Uganda's food insecurity worsens - UN report [Internet]. [cited 2020 Apr 30]. Available from: https://www.independent.co.ug/ugandas-food-insecurity-worsens-un-report/

27. Programme WF. COVID-19 will double number of people facing food crises unless swift action is taken | World Food Programme [Internet]. 2020. Available from: https://www.wfp.org/news/covid-19-will-double-number-people-facing-food-crises-unless-swift-action-taken

28. The World Bank. Food Security and COVID-19 [Internet]. [cited 2020 May 15]. Available from: https://www.worldbank.org/en/topic/agriculture/brief/food-security-and-covid-19

29. United Nations Population Fund. Pandemic and rising tide of violence increase demand for mental

For peer review only

health, psychosocial care [Internet]. Available from: https://www.unfpa.org/news/pandemic-and-rising-tide-violence-increase-demand-mental-health-psychosocial-care

30. Jewkes R, Morrell R. Hegemonic Masculinity , Violence , and Gender Equality : Using Latent Class Analysis to Investigate the Origins and Correlates of Differences between Men. 2018;21(4):547–71.

31. Jewkes R, Morrell R. Gender and sexuality : emerging perspectives from the heterosexual epidemic in South Africa and implications for HIV risk and prevention. 2010;1–11.

32. Cain M, Khanam SR, Nahar S. Class, Patriarchy, and Women's Work in Bangladesh. Popul Dev Rev. 1979 Sep;5(3):405.

33. Muderedzi J, Eide AH, Braathen SH, Stray-Pedersen B, Eide ArneHEide AH, Stine Braathen StineHBraathen sintefno H, et al. Exploring the Relationship Between Food Insecurity, Gender Roles and HIV/AIDS Among Tonga Carers of Disabled Children of Binga in Zimbabwe. Sex Cult. 2019;23:1131–46.

34. Kamali A, Nsubuga RN, Ruzagira E, Bahemuka U, Asiki G, Price MA, et al. Heterogeneity of HIV incidence: a comparative analysis between fishing communities and in a neighbouring rural general population, Uganda, and implications for HIV control. Sex Transm Infect. 2016 Sep 1;92(6):447–54.

35. Uganda Bureau of Statistics (UBOS) and ICF. 2018. Uganda Demographic and Health Survey 2016. Kampala, Uganda and Rockville, Maryland, USA: UBOS and ICF.

36. Statistics UB of. National Population and Housing Census 2014 - Main Report. 2014.

37. Uganda. Ministry of Health. 2003. Uganda national policy guidelines for HIV voluntary counselling and testing. Kampala: Ministry of Health.

38. Ballard, T.J., Kepple, A.W. & Cafiero, C. 2013. The food insecurity experience scale: developing a global standard for monitoring hunger worldwide. Technical Paper. Rome, FAO. (available at http://www.fao.org/economic/ess/ess-fs/voices/en/).

39. Bickel, Gary; Nord, Mark; Price, Cristofer; Hamilton, William; Cook J. Guide to Measuring Household Food Security,; prepared by Bickel, Bickel Gary Mark, Nord Cristofer, Price William, Hamilton and John, Cook. 2000;82.

For peer review only - http://bmjopen.bmj.com/site/about/guidelines.xhtml

40. Roberts TA, Auinger P, Klein JD. Intimate partner abuse and the reproductive health of sexually active female adolescents. J Adolesc Heal. 2005;36(5):380–5.

41. Raj A, Santana MC, La Marche A, Amaro H, Cranston K, Silverman JG. Perpetration of intimate partner violence associated with sexual risk behaviors among young adult men. Am J Public Health. 2006;96(10):1873–8.

42. Mullinax M, Grilo SA, Song XS, Wagman J, Mathur S, Nalugoda F, et al. HIV-Risk Behaviors of Men Who Perpetrate Intimate Partner Violence in Rakai, Uganda. AIDS Educ Prev. 2017 Dec;29(6):527–39.

43. Chirwa ED, Sikweyiya Y, Addo-Lartey AA, Ogum Alangea D, Coker-Appiah D, Adanu RMK, et al. Prevalence and risk factors of physical or sexual intimate violence perpetration amongst men in four districts in the central region of Ghana: Baseline findings from a cluster randomised controlled trial. Dalby AR, editor. PLoS One. 2018 Mar 9;13(3):e0191663.

44. Fleming PJ, Mccleary-sills J, Morton M, Levtov R, Heilman B, Barker G. Risk Factors for Men ' s Lifetime Perpetration of Physical Violence against Intimate Partners : Results from the International Men and Gender Equality Survey (IMAGES) in Eight Countries. 2015;1–18.

45. Palermo T, Bleck J, Peterman A. Tip of the iceberg: Reporting and gender-based violence in developing countries. Am J Epidemiol. 2014;179(5):602–12.

46. Yount KM, Higgins EM, VanderEnde KE, Krause KH, Minh TH, Schuler SR, et al. Men's Perpetration of Intimate Partner Violence in Vietnam. Men Masc. 2016 Apr 24;19(1):64–84.

47. Cardoso LF, Gupta J, Shuman S, Cole H, Kpebo D, Falb KL. What Factors Contribute to Intimate Partner Violence Against Women in Urban, Conflict-Affected Settings? Qualitative Findings from Abidjan, Côte d'Ivoire. J Urban Heal. 2016 Apr 21;93(2):364–78.

48. Lentz EC. Complicating narratives of women's food and nutrition insecurity: Domestic violence in rural Bangladesh. World Dev. 2018 Apr 1;104:271–80.

49. Culbreth R, Swahn MH, Salazar LF, Kasirye R, Musuya T. Intimate Partner Violence and Associated Risk Factors Among Youth in the Slums of Kampala. J Interpers Violence. 2019 Nov 29;088626051988992.

50. Swartout KM. The company they keep: How peer networks influence male sexual aggression. Psychol Violence. 2013;3(2):157–71.

For peer review only

51. Coomber K, Mayshak R, Liknaitzky P, Curtis A, Walker A, Hyder S, et al. The Role of Illicit Drug Use in Family and Domestic Violence in Australia. J Interpers Violence. 2019;

52. Stark L, Seff I, Hoover A, Gordon R, Ligiero D, Massetti G. Sex and age effects in past-year experiences of violence amongst adolescents in five countries. PLoS One. 2019;14(7):1–13.

53. Mckool M, Stephenson R, Winskell K, Tharp T, Parrott D, Arbor A. Case for a Social Norms Approach. 2019;

54. Abramsky T, Devries K, Kiss L, Francisco L, Nakuti J, Musuya T, et al. A community mobilisation intervention to prevent violence against women and reduce HIV/AIDS risk in Kampala, Uganda (the SASA! Study): study protocol for a cluster randomised controlled trial. Trials. 2012;13:96.

55. Promundo, CulturaSalud, and REDMAS (2013). Program P – A Manual for Engaging Men in Father- hood, Caregiving, Maternal and Child Health. Promundo: Rio de Janeiro, Brazil and Washington, D.C. USA. These materials can be reproduced provided credit is given.

56. Lindberg LD et al., Early Impacts of the COVID-19 Pandemic: Findings from the 2020 Guttmacher Survey of Reproductive Health Experiences, New York: Guttmacher Institute, 2020, https://www.guttmacher.org/report/early-impacts-covid-19-pandemic-findings-2020-.

57. World Food Program. COVID-19 Level 3 Emergency: External Situation Report no.17. 2020.

58. Rose DD. Interventions to reduce household food insecurity: a synthesis of current concepts and approaches for Latin America. Rev Nutr. 2008;21(suppl):159s-173s.

59. Bourey C, Williams W, Bernstein EE, Stephenson R. Systematic review of structural interventions for intimate partner violence in low- and middle-income countries: Organizing evidence for prevention Health behavior, health promotion and society. BMC Public Health. 2015;15(1).

Figure 1: Study flow, illustrating the selection process for inclusion into the analysis

Figure 2: Depiction of lifetime prevalence by age group of male lifetime IPV perpetration in two districts of Uganda, May 2018 to July 2019.

For peer review only - http://bmjopen.bmj.com/site/about/guidelines.xhtml

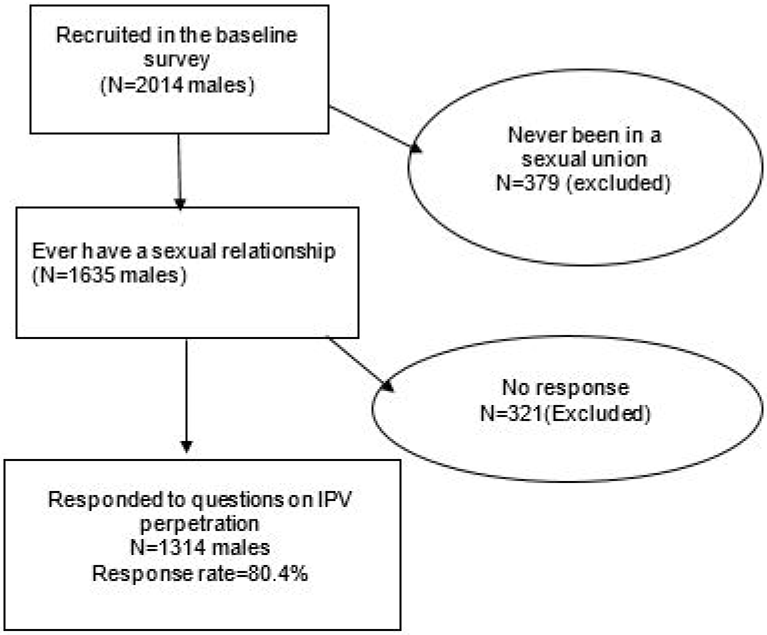

Figure 1: Study flow, illustrating the selection process for inclusion into the analysis

111x88mm (96 x 96 DPI)

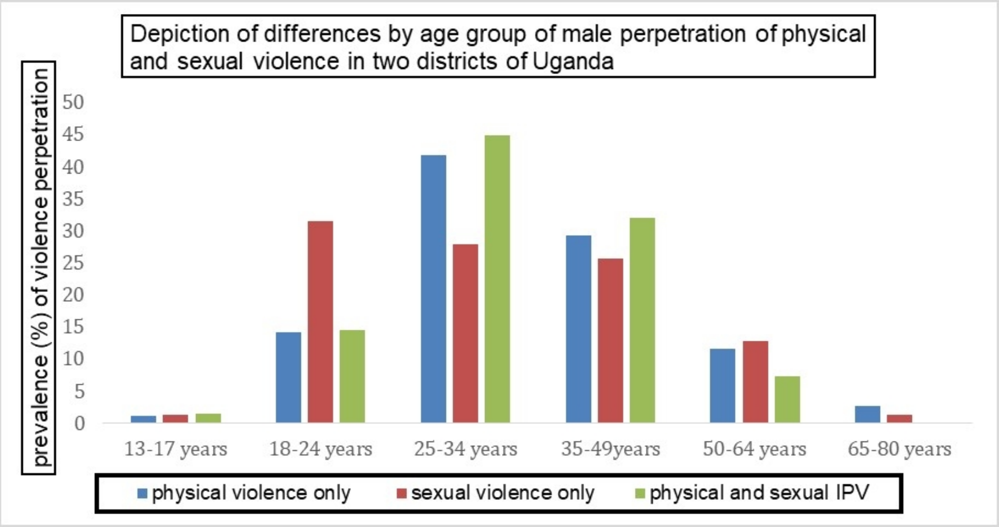

Figure 2: Depiction of lifetime prevalence by age group of male lifetime IPV perpetration in two districts of Uganda, May 2018 to July 2019.

154x81mm (150 x 150 DPI)

Online supplementary Appendix1:

Sensitivity analysis: Characteristics of the analysed sample (N=1314) and non-responders (N=321)

Variable	Analysed sample N (%)	Non-responders N (%)	p-value
**Study Strata**			
Wakiso communities	**578**	**179**	0.364
**Urban -Kazo**	206(35.6)	68(37.9)	
**Semi-urban -Lukwanga**	219(37.9)	62(34.6)	
**Rural -Sentema**	153(26.5)	49 (27.4)	
Hoima communities	**736**	**142**	0.210
**Urban –Ishaka_Kijungu**	243(33.0)	67(47.2)	
**Semi-urban-Butem**	198(25.5)	39(27.5)	
**Rural -Kitoba**	295(40.1)	42 (29.6)	
**Age (mean, SD), interquartile range(years)**	34 ±12.9, 24-42	15.8 ±3.4, 14-17	<0.05
**Education**			
**Primary school and below**	688(52.3)	149(46.4)	>0.001
**Secondary school**	477(36.3)	111(34.5)	
**Post-secondary**	149(11.3)	61(19.0)	
**Not married**	151(11.5)	0	
**Religion**			
**Christianity**	1068(81.3)	230(71.7)	0.02
**Non-Christians**	246(18.7)	91(28.3)	
**HIV status from blood sample**			
**Negative**	1224(94.3)	310(96.6)	<0.001
**Positive**	74(5.7)	11(3.4)	
**Past-year food insecurity**			
**No food insecurity**	995(75.7)	240(74.9)	0.891
**Low food insecurity**	272(20.7)	74(23.2)	
**High food insecurity**	47(3.6)	7(1.9)	

only

STROBE Statement—Checklist of items that should be included in reports of ***cross-sectional studies***

	Item No	Recommendation	Page No
**Title and abstract**	1	(*a*) Indicate the study's design with a commonly used term in the title or the abstract	✓ Page1-2
		(*b*) Provide in the abstract an informative and balanced summary of what was done and what was found	✓ Page 2
**Introduction**			
Background/rationale	2	Explain the scientific background and rationale for the investigation being reported	✓ Page 4-6
Objectives	3	State specific objectives, including any prespecified hypotheses	✓ Page6
**Methods**			
Study design	4	Present key elements of study design early in the paper	✓ Page6-7
Setting	5	Describe the setting, locations, and relevant dates, including periods of recruitment, exposure, follow-up, and data collection	✓ Page7
Participants	6	(*a*) Give the eligibility criteria, and the sources and methods of selection of participants	✓ Page7-8
Variables	7	Clearly define all outcomes, exposures, predictors, potential confounders, and effect modifiers. Give diagnostic criteria, if applicable	✓ Page8-10
Data sources/ measurement	8*	For each variable of interest, give sources of data and details of methods of assessment (measurement). Describe comparability of assessment methods if there is more than one group	✓ *Page9-10*
Bias	9	Describe any efforts to address potential sources of bias	✓ Page7-8
Study size	10	Explain how the study size was arrived at	✓ Page7
Quantitative variables	11	Explain how quantitative variables were handled in the analyses. If applicable, describe which groupings were chosen and why	✓ Page 9-11
Statistical methods	12	(*a*) Describe all statistical methods, including those used to control for confounding	✓ Page 11
		(*b*) Describe any methods used to examine subgroups and interactions	x
		(*c*) Explain how missing data were addressed	x
		(*d*) If applicable, describe analytical methods taking account of sampling strategy	N/A
		(*e*) Describe any sensitivity analyses	✓ Page8
**Results**			
Participants	13*	(a) Report numbers of individuals at each stage of study—eg numbers potentially eligible, examined for eligibility, confirmed eligible, included in the study, completing follow-up, and analysed	✓ Page8
		(b) Give reasons for non-participation at each stage	x
		(c) Consider use of a flow diagram	x

For peer review only - http://bmjopen.bmj.com/site/about/guidelines.xhtml

Descriptive data	14*	(a) Give characteristics of study participants (eg demographic, clinical, social) and information on exposures and potential confounders	✓ Page13
		(b) Indicate number of participants with missing data for each variable of interest	NA
Outcome data	15*	Report numbers of outcome events or summary measures	✓ Page14
Main results	16	(*a*) Give unadjusted estimates and, if applicable, confounder-adjusted estimates and their precision (eg, 95% confidence interval). Make clear which confounders were adjusted for and why they were included	✓ Page15-16
		(*b*) Report category boundaries when continuous variables were categorized	NA
		(*c*) If relevant, consider translating estimates of relative risk into absolute risk for a meaningful time period	NA
Other analyses	17	Report other analyses done—eg analyses of subgroups and interactions, and sensitivity analyses	x
**Discussion**			
Key results	18	Summarise key results with reference to study objectives	✓ Page18
Limitations	19	Discuss limitations of the study, taking into account sources of potential bias or imprecision. Discuss both direction and magnitude of any potential bias	✓ Page20-21
Interpretation	20	Give a cautious overall interpretation of results considering objectives, limitations, multiplicity of analyses, results from similar studies, and other relevant evidence	✓ Page18-20
Generalizability	21	Discuss the generalizability (external validity) of the study results	✓ Page20
**Other information**			
Funding	22	Give the source of funding and the role of the funders for the present study and, if applicable, for the original study on which the present article is based	✓ Page22

*Give information separately for exposed and unexposed groups.

Note: An Explanation and Elaboration article discusses each checklist item and gives methodological background and published examples of transparent reporting. The STROBE checklist is best used in conjunction with this article (freely available on the Web sites of PLoS Medicine at http://www.plosmedicine.org/, Annals of Internal Medicine at http://www.annals.org/, and Epidemiology at http://www.epidem.com/). Information on the STROBE Initiative is available at www.strobe-statement.org.

For peer review only
